# Cell imaging of dopamine receptor using agonist labeling iridium(iii) complex†
†Electronic supplementary information (ESI) available. See DOI: 10.1039/c7sc04798c


**DOI:** 10.1039/c7sc04798c

**Published:** 2017-12-19

**Authors:** Kasipandi Vellaisamy, Guodong Li, Chung-Nga Ko, Hai-Jing Zhong, Sarwat Fatima, Hiu-Yee Kwan, Chun-Yuen Wong, Wai-Jing Kwong, Weihong Tan, Chung-Hang Leung, Dik-Lung Ma

**Affiliations:** a Department of Chemistry , Hong Kong Baptist University , Kowloon Tong , Hong Kong , China . Email: edmondma@hkbu.edu.hk ; Email: dkwong@hkbu.edu.hk; b State Key Laboratory of Quality Research in Chinese Medicine , Institute of Chinese Medical Sciences , University of Macau , Macau , China . Email: duncanleung@umac.mo; c School of Chinese Medicine , Hong Kong Baptist University , Kowloon Tong , Hong Kong , China; d Department of Biology and Chemistry , City University of Hong Kong , Kowloon Tong , Hong Kong , China; e Department of Chemistry , Department of Physiology and Functional Genomics , Center for Research at the Bio/Nano Interface , Shands Cancer Center , UF Genetics Institute , McKnight Brain Institute , University of Florida , Gainesville , USA . Email: tan@chem.ufl.edu; f Molecular Sciences and Biomedicine Laboratory , State Key Laboratory for Chemo/Biosensing and Chemometrics , College of Chemistry and Chemical Engineering , College of Biology , Hunan University , Changsha , China

## Abstract

Dopamine receptor expression is correlated with certain types of cancers, including lung, breast and colon cancers. In this study, we report luminescent iridium(iii) complexes (**11–14**) as intracellular dopamine receptor (D1R/D2R) cell imaging agents. Complexes **11** and **13**, which are conjugated with a dopamine receptor agonist, showed superior cell imaging characteristics, high stability and low cytotoxicity (>100 μM) in A549 lung cancer cells. siRNA knockdown and dopamine competitive assays indicated that complexes **11** and **13** could selectively bind to dopamine receptors (D1R/D2R) in A549 cells. Fluorescence lifetime microscopy demonstrated that complex **13** has a longer luminescence lifetime at the wavelength of 560–650 nm than DAPI and other chromophores in biological fluids. The long luminescence lifetime of complex **13** not only provides an opportunity for efficient dopamine receptor tracking in biological media, but also enables the temporal separation of the probe signal from the intense background signal by fluorescence lifetime microscopy for efficient analysis. Complex **13** also shows high photostability, which could allow it to be employed for long-term cellular imaging. Furthermore, complex **13** could selectively track the internalization process of dopamine receptors (D1R/D2R) in living cells. To the best of our knowledge, complex **13** is the first metal-based compound that has been used to monitor intracellular dopamine receptors in living cells.

## Abstract 2

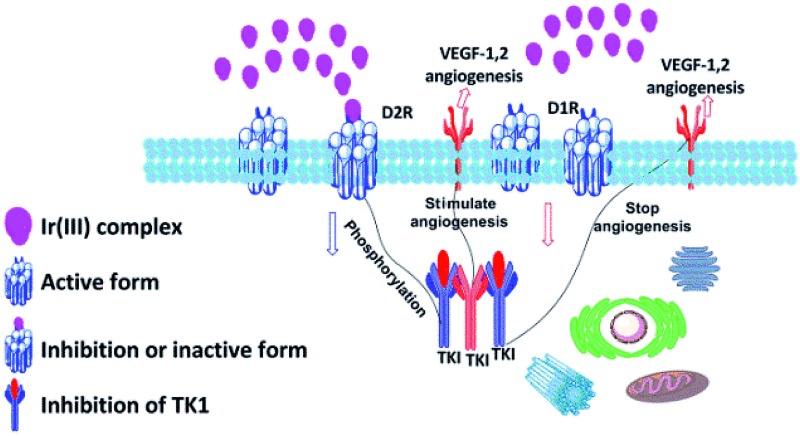
A long-lived complex 13 could selectively bind to dopamine receptors (D1R/D2R) and monitor their internalization in living cells.

## Introduction

Lung cancer is a leading cause of death in both developed and developing countries.^1^ Non-small cell lung cancer (NSCLC) is the most common subtype of lung cancer, with a 5 year survival rate less than 15%.^2^ Angiogenesis, mediated by vascular endothelial growth factor (VEGF) and the VEGF receptor (VEGFR), is important for cancer cell proliferation.^3^ Recently, Basu and coworkers have reported that a dopamine 2 receptor (D2R) agonist is able to inhibit angiogenesis.^4^ Studies with a D2R-knockout mouse model have indicated the molecular mechanism through which D2R/VEGFR-2 crosstalk can mediate the phosphorylation of VEGFR-2. Dopamine receptors also serve as good targets for breast and colon cancers.^5^

Early-stage identification of cancer is a high priority in research.^5^,^6^ Therefore, the development of methods for the detection of cancer biomarkers in living systems has attracted tremendous interest.^7^–^9^ Studies have shown that dopamine receptor (D1R/D2R) expression is higher in NCI-H69 NSCLC cells.^10^ Previously, the expression of the dopamine (D1/D2) receptor in these cells has been monitored using an iodosulpride isotope probe^11^ which is subjected to biases caused by the incubation of isotope. Fluorescent probes^12^–^14^ and biotin derivatives^15^ of dopamine agonists have also been developed as probes of D1R and D2R, whereas nitrobenzoxadiazole (NBD) and fluorescein-coupled dopamine agonists have been applied in the fluorescent labelling of D1R and D2R sites in monkey pituitary gland. However, these probes are generally limited by their short luminescence lifetimes (1–10 ns) and the phenomenon of self-quenching, photobleaching and fluorescence resonance energy transfer (FRET). Although radiolabelled probes for dopamine receptors include S-labeled cRNA probes and [^123^I] N_3_-NAP have been extensively reported,^16^–^21^ great caution is required when handling these radioactive materials. To the best of our knowledge, metal complex probes capable of identifying dopamine receptors (D1R and D2R) in living cells have not yet been developed. In particular, the long luminescence lifetime, large Stokes shift, high luminescence quantum yield and high photostability properties of iridium(iii) complexes render them an excellent alternative agent for dopamine tracking.^30^,^31^

**Scheme 1:**
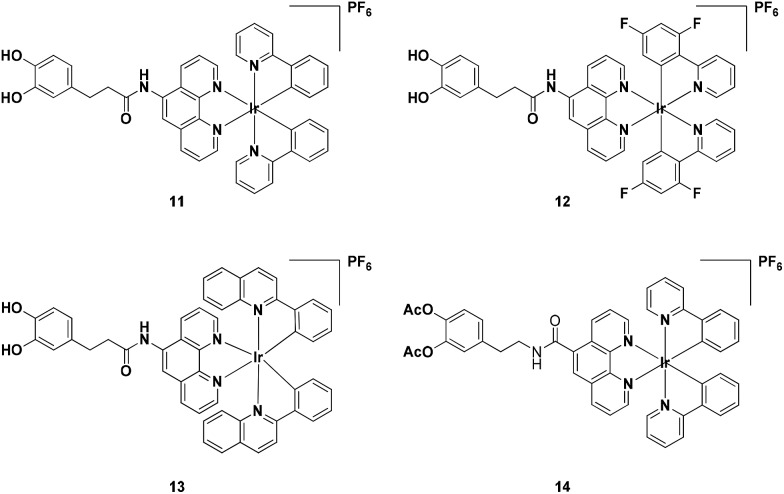
Iridium(iii) dopamine complexes as D1R and D2R probes.

In this study, four cyclometalated iridium(iii) complexes **11–14** with general structure [Ir(N–C)_2_(N–N)](PF_6_) (where N–N = 3-(3,4-dihydroxyphenylagonistsagonists)-*N*-(1,10-phenanthrolin-5-yl) propanamide (**6**) or 4-(2-(1,10-phenanthroline-5-carboxamido)ethyl)-1,2-phenylene diacetate (**9**) and N–C = 2-phenylpyridine (ppy), 2-(2,4-difluorophenyl)pyridine (dfppy), or 2-phenylquinoline (pq)) were designed and synthesised (Scheme 1). As ligands **6** and **9** are derived from dopamine agonists, we hypothesized that the conjugated complexes would be able to effectively recognize dopamine receptors (D2R/D1R). Notably, complexes **11** and **13**, showed superior cell imaging characteristics, high stability and low cytotoxicity (>100 μM) in A549 lung cancer cells. siRNA knockdown and dopamine competitive assays indicated that complexes **11** and **13** could selectively bind to dopamine receptors (D1R/D2R) in A549 cells. Furthermore, complex **13** possesses useful photophysical properties including long luminescence lifetimes, high photostability and high luminescence quantum yield. Since most of the background fluorescence in cell medium has a luminescence lifetime of less than 3 ns, the relatively longer luminescence lifetime for complex **13** should enable the temporal separation of the probe signal from the intense background signal by fluorescence lifetime microscopy. To the best of our knowledge, this is the first application of iridium(iii) complexes for imaging D2R/D1R within living cells.

## Results and discussions

### Synthesis of N^N ligands **6** and **9**

We envisaged that conjugating dopamine agonists, such as dopamine or 3-(3,4-dihydroxyphenyl)propanoic acid (**1**),^22^ to an iridium(iii) scaffold could generate effective probes for dopamine receptors (D1R and D2R). In our designed strategy, the dopamine agonists were conjugated to N^N ligands **6** and **9**.

To synthesize ligand **6**, compound **1** was first converted to the corresponding methyl ester (**2**) in acidic conditions using MeOH as solvent (Scheme S1†).^23^–^25^ Compound **2** was protected as the tetrahydropyranyl ethers (THP) using pyridinium *p*-toluenesulfonate (PPTS) in dry DCM to generate compound **3** in 62% yield.^26^ Subsequently, the THP-protected methyl ester (**3**) was selectively hydrolyzed using LiOH to furnish the corresponding THP-protected acid (**4**) after acidic workup. Compound **4** was then coupled with 1,10-phenanthrolin-5-amine using standard 1-ethyl-3-(3-dimethylaminopropyl)carbodiimide (EDCI) coupling to generate compound **5**. Finally, THP deprotection with a catalytic amount of PPTS in EtOH produced ligand **6** with a yield of 94%. Ligand **6** was characterized by NMR spectroscopy and HRMS (Fig. S1 and S2†).

Ligand **9** was generated in 62% yield from an acetylated dopamine derivative (**8**) and 1,10-phenanthroline-5-carboxylic acid (**7**) using EDCI coupling (Scheme S2†). Ligand **9** was characterized by NMR spectroscopy and HRMS (Fig. S9 and S10†).

### Iridium(iii) complexes synthesis

Complexes **11–14** were synthesised in good yields (85–91%) by reacting two equivalents of N^N ligands (**6** or **9**) and the corresponding cyclometalated iridium(iii) dimers in CH_2_Cl_2_/CH_3_OH (1 : 1, v/v), followed by chloride anion exchange with NH_4_PF_6_. Complexes **11–14** were purified by silica gel column chromatography and characterized by ^1^H, ^13^C NMR spectroscopy and MALDI-HRMS. The photophysical properties of complexes **11–14**, including their luminescence quantum yields, emission properties and UV-vis absorption properties were measured in ACN and are reported in Table S1.† Excitation of **13** (10 μM) at 334 nm produced a maximum emission at 558 nm which is assigned to the metal-to-ligand charge-transfer (MLCT) state and is typical for iridium(iii) complexes. Complex **13** also displays large Stokes shift of 215 nm, which is considerably larger than those generally displayed by organic molecules and can efficiently prevent self-quenching. UV-vis absorption spectra of complexes **11–14** are presented in Fig. S13.† Complex **13** exhibited an intense absorption at 255 and 280 nm and a moderate peak at 331 and 446 nm in CH_2_Cl_2_. The bands are assigned to the promotion of electrons based on the ligand-centered (π–π*) transition and the metal-to-ligand charge transfer (MLCT) transition respectively. Furthermore, complexes **13** and **14** were both stable in a DMSO-*d*_6_ and D_2_O mixture (9 : 1) at 25 °C for seven days, as revealed by NMR spectroscopy, indicating that the acetyl groups of **14** are sufficiently stable to hydrolysis under ambient conditions (Fig. S14 and S15†). Among the four complexes, complex **13** showed the highest luminescence quantum yield of 0.245 and a long luminescence lifetime of 4.61 μs. Complexes **11** and **14** also showed long luminescence lifetimes of 4.36 and 4.65 μs respectively. In contrast, complex **12** was non-emissive. The long luminescence lifetimes exhibited by iridium(iii) complexes **11**, **13** and **14** could enable their emission to be distinguished from background fluorescence by the use of time-resolved emission spectroscopy (TRES).

**Fig. 1:**
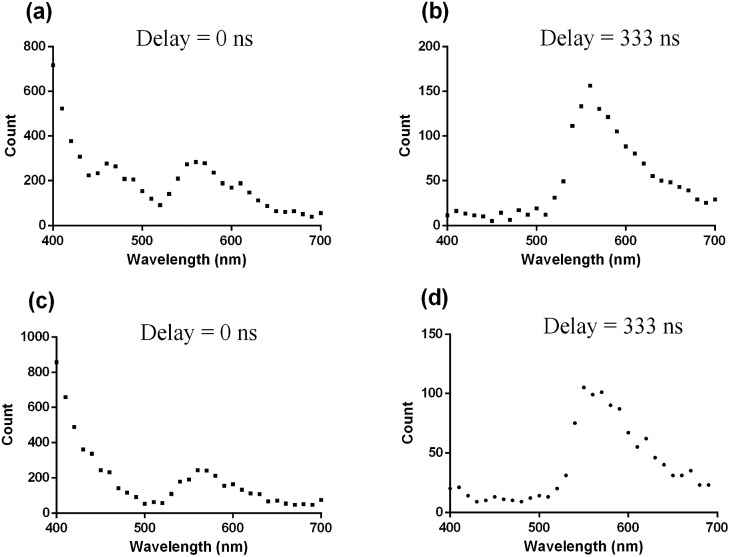
Time-resolved fluorescence emission spectra of complex **13** in PBS buffer (pH = 7.0, 25 °C) in the presence of Cm-460 at (a) delay = 0 ns and (b) delay = 333 ns or with THS at (c) delay = 0 ns and (d) delay = 333 ns. The peak of complex **13** was partially obscured by the emission peaks of the fluorescent media when delay = 0 ns, while the peak of complex **13** became more evident when delay = 333 ns.

To validate this hypothesis, coumarin 460 (Cm-460) or thioflavin S (THS) was employed as model matrix interferents. In contrast to the iridium(iii) complexes, organic fluorophores typically show nanosecond luminescence lifetimes. When the luminescence spectra were recorded directly after the excitation pulse without any delay (*λ*_exc_ = 355 nm), Cm-460 exhibited a strong emission peak at 455 nm while THS exhibited a moderate peak at 540 nm. Therefore, the peak of complex **13** was partially obscured by the trailing edge and the overlapping emission peaks of Cm-460 and THS, respectively (Fig. 1a and c). In contrast, when the luminescence spectra were recorded with a delay of 333 ns after the excitation pulse, the short-lived fluorescence of Cm-460 and THS were eliminated, and the emission of complex **13** became more evident (Fig. 1b and d).

### Cytotoxicity and cell staining in A549 cells

In consideration of the promising luminescent behaviour shown by transition-metal complexes, the cytotoxicity of complexes **11–14** was measured in A549 cells, a human NSCLC cell line with dopamine receptor expression, using the MTT (3-(4,5-dimethylthiazol-2-yl)-2,5-diphenyltetrazolium bromide) assay. The results revealed that complexes **11–13** exhibited IC_50_ values above 100 μM, whereas complex **14** showed an IC_50_ value of 70.79 μM (Fig. S16†). This indicates that all of complexes are relatively nontoxic to cells, making them suitable for cell staining experiments.

**Fig. 2:**
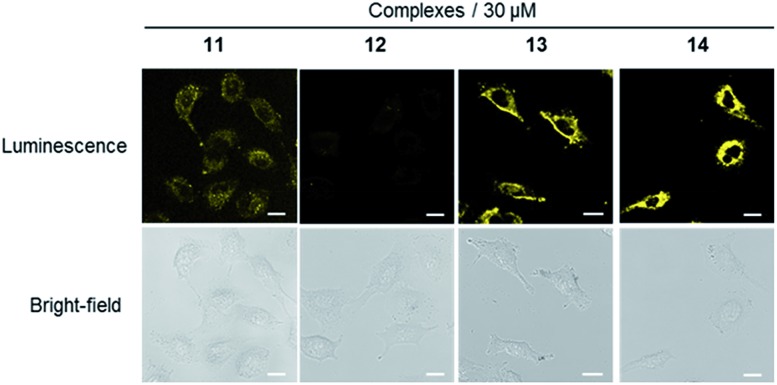
Luminescence and bright-field images of A549 cells stained with 30 μM of complexes **11–14** for 1 h. Scale bar = 15 μm.

We next investigated the application of the iridium(iii) complexes for cell staining. A549 cells were incubated with complexes **11–14** (30 μM) for 1 h and then washed with phosphate buffer. Luminescence imaging using a confocal laser scanning microscope with excitation at 488 nm revealed that complexes **11**, **13** and **14** showed strong luminescence in A549 cells (Fig. 2), with luminescence intensity increasing with complex concentration (Fig. S18–S20†). In contrast, minimal luminescence was observed with complex **12**, even when the concentration of the complex was increased to 60 μM (Fig. S17†). We presume that complexes **11**, **13** and **14** could interact strongly with the dopamine receptor (D1R/D2R) *via* their dopamine agonist moieties, thereby leading to an enhanced luminescence of the cell.

### Validation of dopamine receptor binding in A549 cells

**Fig. 3:**
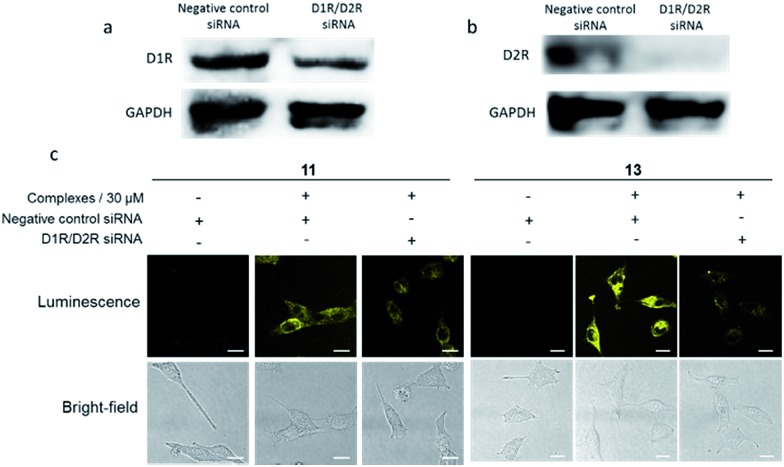
(a) The relative amount of D1R in A549 cells before or after D1R/D2R siRNA stimulation. (b) The relative amount of D2R in A549 cells before or after D1R/D2R siRNA stimulation. (c) Luminescence and bright-field images of A549 cells with D1R/D2R knockdown using siRNA. A549 cells were stained with or without complexes **11** and **13** (30 μM) for 1 h. Scale bar = 15 μm.

In order to verify that the complexes were interacting with dopamine receptors (D1R/D2R) in cells, siRNA knockdown experiments were performed with D1R/D2R siRNA in A549.^27^–^29^ As shown by Western blotting experiments, the levels of both D1R (Fig. 3a) and D2R (Fig. 3b) were reduced significantly in the presence of D1R/D2R siRNA, with a greater reduction for D2R. Next, complexes **11** and **13** were introduced into D1R/D2R knockdown cells. As depicted in Fig. 3c, the luminescence of complexes **11** and **13** was reduced significantly, indicating that the emission enhancement of the complexes required the presence of dopamine receptors in living cells. On the other hand, the luminescence intensity of complex **14** was relatively unaffected in the presence of D1R/D2R siRNA (Fig. S21†). This suggests that complex **14** may show nonspecific binding to molecules other than dopamine receptors in the intracellular environment.

**Fig. 4:**
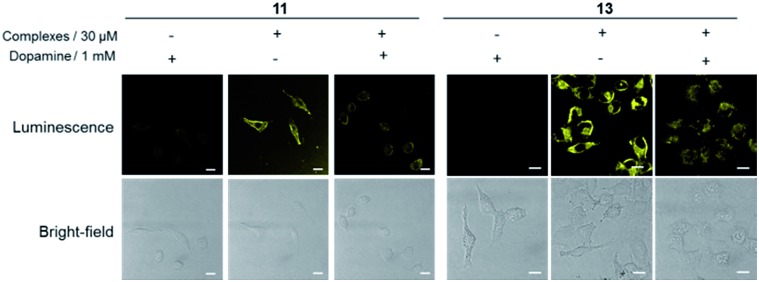
Luminescence and bright-field images of A549 cells. A549 cells were pretreated with or without dopamine (1 mM) for 1 h, followed by staining with complexes **11** and **13** (30 μM) for 1 h. Scale bar = 15 μm.

To provide further evidence that complexes **11** and **13** interacted with dopamine receptors (D1R/D2R) in living cells, we preincubated A549 cells with an excess of dopamine (1 mM) for 1 h, before staining with complexes **11** and **13** (30 μM) for 1 h. Interestingly, the luminescence intensity of complexes **11** and **13** was decreased in the presence of dopamine (Fig. 4), suggesting that the presence of dopamine blocks the interaction between complexes and dopamine receptors (D1R/D2R) in living cells, presumably *via* competitive binding to dopamine receptors. Overall, complex **13** showed the highest luminescence enhancement in the cell imaging experiments, indicating that it could serve as a useful scaffold for developing probes for dopamine receptors in living cells.

### Photostability of **13***in cellulo*

**Fig. 5:**
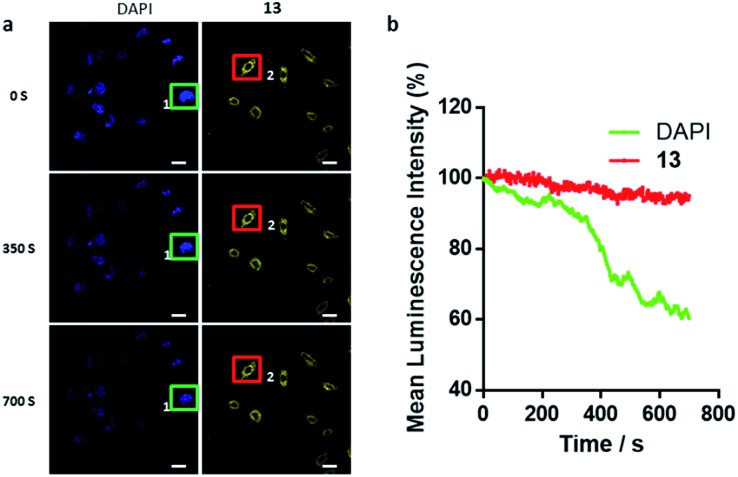
Comparison of DAPI and **13** for resistance to photobleaching. (a) Confocal luminescence images of fixed A549 cells stained with DAPI and **13** under continuous irradiation at 405 nm with different laser scan times (0, 350, 700 s). Scale bar = 30 μm. (b) The relative mean luminescence intensity of DAPI and **13** collected from region 1 (green, 430 to 480 nm) and 2 (red, 550 to 650 nm).

Considering that high photostability is very important for the practical application of a cellular probe for bioimaging, a photobleaching assay was performed with iridium(iii) complex **13** in paraformaldehyde-fixed A549 cells. 4′,6-Diamidino-2-phenylindole (DAPI), an organic and commercial dye for staining nuclei in cells, was used as a benchmark for photostability. After continuous excitation at 405 nm for 700 s, the mean luminescence intensity of complex **13** (550–650 nm, region 2) only decreased by 4.9%. Whereas the luminescence of DAPI (430–480 nm, region 1) decreased by 39.6% (Fig. 5). This result demonstrates that **13** exhibits higher photostability compared to DAPI, indicating that **13** could be employed as a potential bioimaging luminescent probe for continuous tracking studies over a long period of time.

### Complex **13** could selectively monitor the internalization of dopamine receptors *in cellulo*

**Fig. 6:**
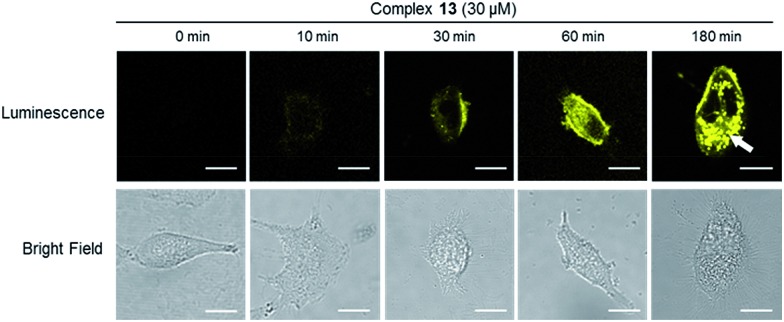
A549 cells were stained by complex **13** for 0, 10, 30, 60 and 180 min. The white arrow indicates the punctuated pattern. Scale bar = 15 μm.

The internalization of dopamine receptor is important for maintaining homeostatic control in the cell. We therefore explored the application of complex **13** to track the internalization of dopamine receptors (D1R/D2R) in lung cancer cells. The intracellular luminescence intensities of A549 cells treated with complex **13** (30 μM) for different times (0, 10, 30, 60 and 180 min) were monitored by using fluorescent microscopy. The results showed that luminescence of the cells increased over time and was predominantly localized in the cell boundaries within 60 min (Fig. 6). However, at 180 min, the luminescence of cell boundaries was significantly reduced while that of the cytoplasm was enhanced in a punctuated pattern (Fig. 6), indicating that complex **13** may be an agonist of dopamine receptors and has the potential to monitor the internalization of D1/D2-receptors in A549 cells. This result was also confirmed by assessing the luminescence in D1R/D2R knockdown A549 cells after 180 min incubation, which showed a decrease in the luminescence of the cytoplasm as expected (Fig. S22†). Collectively, these results suggest that complex **13** could not only target dopamine receptors (D1R/D2R), but also monitor the internalization of dopamine receptors (D1R/D2R) in living cells.

### Lifetime imaging

**Fig. 7:**
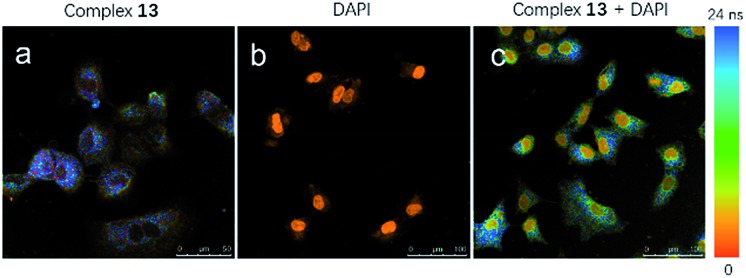
Fluorescence lifetime confocal images of A549 cells treated with (a) complex **13**, (b) DAPI and (c) complex **13** + DAPI. The excitation light of 800 nm from the MP laser (80 MHz pulse repetition rate) was focused onto the sample with a 40× objective lens for two-photon excitation. The luminescence signals were collected with the range of 550–650 nm.

Considering that the long luminescence lifetime of transition metal complexes could overcome issues of an endogenous fluorescent background signal, **13**-stained A549 cells were visualized with fluorescence lifetime imaging microscopy (FLIM) to demonstrate the merits of **13** in bioimaging. Most of the background fluorescence has a luminescence lifetime of less than 3 ns, which is in contrast to the long luminescence lifetimes of iridium(iii) complexes. This large difference enables the temporal separation of the probe fluorescence signal from the intense background signal in cell media. As shown in Fig. 7a, a long luminescence lifetime about 22 ns of complex **13** in cell boundaries and the cytoplasm (blue) was detected by using a Leica TCS SP5 confocal laser scanning microscope system in A549 cells after 1 h incubation. An MP laser with two-photon excitation light of 800 nm was used for excitation. The FLIM images consisted of 512 × 512 pixels were scanned with a scan speed of 400 Hz. In contrast, DAPI only had a short luminescence lifetime in the cell nucleus (orange) in which its signal decay in 2.1 ns after the pulsed excitation (Fig. 7b). To validate the potential of **13** in bioimaging, FLIM imaging of A549 cells co-incubated with **13** and DAPI was also performed. A short excitation pulse with 80 MHz repetition rate excites all chromophores in the cell that absorb at the excitation wavelength, including complex **13** and DAPI in the cell medium. As observed from Fig. 7c, complex **13** showed a longer apparent lifetime than DAPI in A549 cells (scale range from 0 ns (red) to 24 ns (blue)), and displayed a longer fluorescence intensity within cell boundaries and the cytoplasm than that of DAPI in the cell nucleus (orange). The FLIM results indicate that **13** has a longer apparent lifetime within the cell than DAPI, suggesting that **13** is suitable for the long-lived luminescence imaging even in the presence of an endogenous fluorescence background signal.

## Experimental

### Synthesis of 3,4-dihydroxyphenylacetic acid methyl ester (**2**)^1^

A solution of 1 g (0.0054 mol) of 3,4-dihydroxyphenylacetic acid (**1**) in 50 mL of MeOH containing 1 mL of concentrated sulphuric acid (H_2_SO_4_) was refluxed overnight. The completion of reaction was monitored by TLC. After completion, the MeOH was evaporated, and the residue was dissolved in 50 mL of EtOAc. The ethyl acetate layer was washed with dilute NaHCO_3_, water, and brine, followed by drying with Na_2_SO_4_. The organic layer was evaporated to give 1.2 g of the ester **2** (97%) that was used in the next step without further purification.

### Synthesis of 3,4-bis-(tetrahydropyranyloxy)phenylacetic acid methyl ester (**3**)

A solution of 1 g (5.1 mmol) of **2** and 120 mg (0.102 mmol) of pyridinium *p*-tosylate in 30 mL of DCM was treated dropwise with 4.5 mL (5.1 mmol) of THP in 5 mL of DCM. The mixture became homogeneous after about 1 h, and the reaction was essentially complete. When the TLC indicated the absence of starting material, the reaction mixture was washed twice with water, dried (Na_2_SO_4_), and evaporated to give 1.8 g of **3** (98%) as a mixture of diastereoisomers, which were used in the next step without further purification.

### Synthesis of 3,4-bis-(tetrahydropyranyloxy)phenylacetic acid (**4**)

Compound **3** (1 g, 2.74 mmol) was dissolved in 20 mL of THF/water (1 : 1) mixture at 0 °C, followed by the addition of LiOH (3.2 mmol), and allowed to react for 1 h. When the TLC indicated the absence of starting material, the pH of the reaction mixture was adjusted to 4–5 using 5% HCl, extracted with ethyl acetate, and evaporated to give 0.9 g of **4** (98%) as a mixture of diastereoisomers, which were used in the next step without further purification.

### Synthesis of 3-(3,4-dihydroxyphenyl)-*N*-(1,10-phenanthrolin-5-yl)propanamide (**6**)

5-Amino-1,10-phenanthroline (0.5 g, 2.5 mmol, 1 eq.) was dissolved in distilled DCM (50 mL) at 0 °C. Compound **4** (0.8 g, 2.5 mmol, 1 eq.) was added, followed by EDCI (0.95 g, 5 mmol, 2 eq.) and, finally, 4-dimethylaminopyridine (DMAP) (0.3 g, 2.3 mmol, 1 eq.). After complete addition of reagents, the reaction mixture was stirred at 0 °C for a further 1 h and allowed to complete for 24 h. Finally, the solvent was removed under reduced pressure, and the red colour residue was dissolved in EtOAc. The organic layer was washed with NaHCO_3_ (saturated) solution and dried over Na_2_SO_4_. The organic layer was evaporated and purified by column chromatography to get compound **5** (0.9 g, 75%). Compound **5** was dissolved (0.50 g, 0.94 mmol) in EtOH (10 mL), and PPTS was added (4 mg, 0.002 mmol). The resulting mixture was stirred at 55 °C for 6 h. After removal of EtOH under vacuum, the residue was washed with EtOAc and ether to give a pure white solid compound **6** (0.32 g, 94%). ^1^H NMR (400 MHz, DMSO) *δ* 9.12 (dd, *J* = 4.2, 1.5 Hz, 1H), 9.05 (dd, *J* = 4.3, 1.7 Hz, 1H), 8.46 (dd, *J* = 8.2, 1.7 Hz, 1H), 8.34 (dd, *J* = 8.4, 1.6 Hz, 1H), 8.11 (s, 1H), 7.76 (m, 2H), 6.67 (m, 2H), 6.53 (d, *J* = 8.0 Hz, 1H), 2.83 (d, *J* = 6.3 Hz, 2H), 2.77 (d, *J* = 5.9 Hz, 2H). ^13^C NMR (101 MHz, DMSO) *δ* 171.71, 149.75, 149.36, 145.76, 145.22, 137.42, 135.75, 131.77, 129.94, 127.47, 124.78, 123.51, 122.74, 120.36, 118.87, 115.94, 115.53, 37.98, 30.56. HRMS: calcd for C_21_H_17_N_3_O_3_: 359.3780 found [M + 1]^+^: 360.1609.

### Synthesis of 4-(2-(1,10-phenanthroline-5-carboxamido)ethyl)-1,2-phenylene diacetate (**9**)

4-(2-Aminoethyl)-1,2-phenylene diacetate (0.7 g, 3.12 mmol, 1 eq.) was dissolved in distilled DCM (30 mL) at 0 °C. Compound **7** (1.28 g, 4.68 mmol, 1.5 eq.) was added, followed by 1-ethyl-3-(3-dimethylaminopropyl)carbodiimide (EDCI) (0.95 g, 5 mmol, 2 eq.), HOBt (0.42 g, 3.12 mmol, 1 eq.), and, finally, Et_3_N (0.63 g, 6.3 mmol, 2 eq.). After complete addition of reagents, the reaction mixture was stirred at 0 °C for a further 1 h and allowed to complete for 24 h. Completion of reaction was monitored by TLC using MeOH/DCM (10 : 90). The solvent was removed under reduced pressure, and the red colour residue was dissolved in EtOAc. The organic layer was washed with NaHCO_3_ (saturated) solution and dried over Na_2_SO_4_. The organic layer was evaporated and purified by column chromatography to get compound **9** (0.5 g, 62%). ^1^H NMR (400 MHz, CDCl_3_) *δ* 9.01 (d, *J* = 3.0 Hz, 2H), 8.49 (dd, *J* = 8.4, 1.5 Hz, 1H), 8.08 (dd, *J* = 8.1, 1.4 Hz, 1H), 7.57 (s, 1H), 7.50 (ddd, *J* = 10.3, 8.3, 4.3 Hz, 2H), 7.20 (s, 1H), 7.12 (m, 3H), 6.75 (s, 1H), 3.81 (dd, *J* = 12.7, 6.4 Hz, 2H), 3.03 (t, *J* = 6.6 Hz, 2H), 2.25 (s, 3H), 2.22 (s, 3H). ^13^C NMR (101 MHz, CDCl_3_) *δ* 168.45, 168.40, 168.03, 151.25, 150.35, 142.13, 140.77, 137.77, 136.76, 134.41, 132.87, 127.03, 126.93, 125.72, 125.57, 124.10, 123.69, 123.49, 123.42, 40.86, 34.84, 20.75, 20.71. HRMS: calcd for C_25_H_21_N_3_O_5_Na: 466.1373 found [M + Na]^+^: 466.1352.

### Materials and cell lines

All chemicals were purchased from Sigma-Aldrich and were used as received. Lipofectamine™ 3000 reagent was purchased from Invitrogen (Carlsbad, CA, USA). Fetal bovine serum (FBS) and Dulbecco's Modified Eagle's Medium (DMEM) were purchased from Gibco BRL (Gaithersburg, MD, USA).

### Cell viability assay

A549 cells were seeded at the density of 5000 cells per well in 96 well plates and incubated for 12 h. Complexes dissolved in DMSO were added to cells at indicated concentrations for 48 h, respectively. Then 10 μL of 5 mg mL^–1^ MTT (3-(4,5-dimethylthiazol-2-yl)-2,5-diphenyltetrazolium bromide) reagent were added to each well. After 4 h incubation in the dark, 100 μL of DMSO were added to each well, and the intensity of absorbance was determined by a SpectraMax M5 microplate reader at a wavelength of 570 nm.

### Cell imaging

A549 cells were seeded into a glass-bottomed dish (35 mm dish with 20 mm well). After 24 h, the cells were incubated with complexes for indicated time periods or concentrations and then washed with phosphate-buffered saline three times. The luminescence imaging of complexes in cells was carried out by a Leica TCS SP8 confocal laser scanning microscope system. The excitation wavelength was 488 nm.

### Immunoblotting

A549 cells were harvested in lysis buffer after knockdown treatment, and the protein concentration was determined by using the BCA assay. Total proteins were separated on SDS-polyacrylamide gel electrophoresis and then transferred onto polyvinylidene difluoride membranes (Millipore). After 1 h incubation with blocking buffer at room temperature, membranes were incubated with the primary antibodies at 4 °C overnight and the secondary antibodies for 1 h incubation at room temperature. The protein bands were then stained by ECL Western Blotting Detection Reagent (GE Healthcare) and visualized using the ChemiDoc™ MP Imaging System.

### Dopamine D1/D2 receptor knockdown assay

A549 cells were seeded in 6 well plates at about 80% confluence in DMEM for 12 h. Lipofectamine™ 3000 reagent and siRNA were gently mixed in FBS-free DMEM medium. After 15 min incubation at room temperature, 500 μL of siRNA-lipid complex were directly added to cells in 1.5 mL DMEM culture medium. Then, A549 cells were incubated at 37 °C in a CO_2_ incubator for 48 h before use.

### Photobleaching assay and FLIM imaging

A549 cells were seeded into a confocal glass-bottomed dish (35 mm dish with 20 mm well) and incubated at 37 °C for 12 h. Subsequently, complex **13** (30 μM) was added and the wells were further incubated for 1 h. Before cell imaging, cells were pre-fixed with 4% paraformaldehyde for 15 min, followed by washing three times with phosphate-buffered saline. DAPI staining solution was added and the wells were incubated for 3 min. After washing in phosphate-buffered saline, fluorescence imaging was carried out with continuous excitation (*λ* = 405 nm) for 700 s using a Leica TCS SP8 confocal laser scanning microscope. For FLIM imaging, complex **13** (60 μM) was added and the wells were incubated for 1 h. The cells were pre-fixed with 4% paraformaldehyde for 15 min, followed by washing three times with phosphate-buffered saline. DAPI staining solution or buffer was added and the wells were further incubated for 3 min, followed by imaging using a Leica TCS SP5 confocal laser scanning microscope with a 40× objective lens. An MP laser with 800 nm two-photon excitation wavelength and 80 MHz repetition rate were used for excitation. The luminescence signals were collected in the range of 550–650 nm. Photoluminescence lifetime images with 512 × 512 pixels were acquired with scan speed of 400 Hz. The images were recorded after an excitation pulse without any time delay. Finally, the FLIM data were analyzed using a pixel-based fitting software (SPCImage, Becker & Hickl). An incomplete decay model in SPCImage software was employed for the calculation of lifetime for complex **13**.

## Conclusions

Four iridium(iii) complexes (**11–14**) bearing dopamine or 3-(3,4-dihydroxyphenyl)propanoic acid as dopamine agonists were synthesized. Among the synthesized complexes, complexes **11** and **13** displayed superior photophysical characteristics and high stability in living cells. Moreover, D1R/D2R siRNA knockdown and dopamine competition experiments suggested that complexes **11** and **13** could selectively bind to dopamine receptors (D1R/D2R) in living cells. Finally, the photostable complex **13** could also be used to monitor the internalization of dopamine receptors (D1R/D2R) in living cells, and its application for long-lived luminescence imaging even in the presence of endogenous fluorescence background signal was also demonstrated using FLIM. We envisage that these complexes could serve as useful scaffolds for the development of luminescent dopamine receptors cell imaging probes.

## Conflicts of interest

There are no conflicts to declare.

## Supplementary Material

Supplementary information
