# Supplementary material for: Cell imaging of dopamine receptor using agonist labeling iridium(iii) complex
Source: Chem Sci. 2017 Dec 19;9(5):1119–25. doi: 10.1039/c7sc04798c (PMC5885777; doi:10.1039/c7sc04798c)
Supplement: Supplementary file 1 [file SC-009-C7SC04798C-s001.pdf]

Electronic Supplementary Material (ESI) for Chemical Science.
This journal is © The Royal Society of Chemistry 2017

Supporting Information

Cell imaging of dopamine receptor using agonist labeling iridium(III) complex

Kasipandi Vellaisamy,[a†] Guodong Li,[b†] Chung-Nga Ko,[a†] Hai-Jing Zhong,[b] Sarwat Fatima,[c] Hiu-Yee Kwan,[c] Chun-Yuen Wong,[d] Wai-Jing Kwong,*[a] Weihong Tan,*[e,f], Chung-Hang Leung,*[b] Dik-Lung Ma*[a]

a Department of Chemistry, Hong Kong Baptist University, Kowloon Tong, Hong Kong, China. E-mail: edmondma@hkbu.edu.hk, dkwong@hkbu.edu.hk

b State Key Laboratory of Quality Research in Chinese Medicine, Institute of Chinese Medical Sciences, University of Macau, Macau, Chin. E-mail: duncanleung@umac.mo

c School of Chinese Medicine, Hong Kong Baptist University, Kowloon Tong, Hong Kong, China

d Department of Biology and Chemistry, City University of Hong Kong Kowloon Tong, Hong Kong (China)

e Department of Chemistry and Department of Physiology and Functional Genomics, Center for Research at the Bio/Nano Interface, Shands Cancer Center, UF Genetics Institute, McKnight Brain Institute, University of Florida, Gainesville, USA. E-mail: tan@chem.ufl.edu

f Molecular Sciences and Biomedicine Laboratory, State Key Laboratory for Chemo/Biosensing and Chemometrics, College of Chemistry and Chemical Engineering and College of Biology, Hunan University, Changsha, China

Material

General Experiment

Mass spectrometry was performed at the Mass Spectroscopy Unit at the Department of Chemistry, Hong Kong Baptist University, Hong Kong (China). Deuterated solvents for NMR purposes were obtained from Armar and used as received. ^{1}H and ^{13}C NMR were recorded on a Bruker Avance 400 spectrometer operating at 400 MHz (^{1}H) and 100 MHz (^{13}C). ^{1}H and ^{13}C chemical shifts were referenced internally to solvent shift ($CDCl_3$, $MeOD_4$, DMSO-d6, acetone-d6: ^{1}H, 2.05, ^{13}C, 29.8). Chemical shifts are quoted in ppm, the downfield direction being defined as positive. Uncertainties in chemical shifts are typically ±0.01 ppm for ^{1}H and ±0.05 for ^{13}C.

Coupling constants are typically ±0.1 Hz for ^1H-^1H and ±0.5 Hz for ^1H-^{13}C couplings. The following abbreviations are used for convenience in reporting the multiplicity of NMR resonances: s, singlet; d, doublet; t, triplet; q, quartet; m, multiplet; br, broad. All NMR data were acquired and processed using standard Bruker software.

Photophysical measurement

Lifetime measurements for complex **13** were performed on a PTI TimeMaster C720 Spectrometer (Nitrogen laser: pulse output 335 nm) fitted with a 395 nm filter. All solvents used for the lifetime measurements were degassed using three cycles of freeze-vac-thaw. Luminescence quantum yields were determined using the method of Demas and Crosby with $[Ru(bpy)_3][PF_6]_2$ in degassed acetonitrile as a standard reference solution (Φr = 0.062) and were calculated according to the following reported equation:

$$\Phi_S = \Phi_r(B_r/B_S)(n_S/n_r)^2(D_S/D_r) \quad (1)$$

where the subscripts s and r refer to the sample and reference standard solution respectively, n is the refractive index of the solvents, D is the integrated intensity, and Φ is the luminescence quantum yield. The quantity B was calculated by $B = 1 - 10^{-AL}$, where A is the absorbance at the excitation wavelength and L is the optical path length.

Complex 11. (Yield: 91%) ^{1}H NMR (400 MHz, $CDCl_3$) δ 8.37 (s, 1H), 8.35 (d, J = 1.3 Hz, 1H), 8.33 (d, J = 1.3 Hz, 1H), 8.19 (s, 1H), 8.09 (td, J = 4.9, 1.3 Hz, 2H), 7.86 (d, J = 8.0 Hz, 2H), 7.83 (d, J = 8.3 Hz, 2H), 7.65 (d, J = 1.7 Hz, 2H), 7.63 (d, J = 2.5 Hz, 2H), 7.61 (d, J = 3.2 Hz, 1H), 7.05 (s, 1H), 7.03 (d, J = 5.1 Hz, 1H), 7.01 (d, J = 5.0 Hz, 1H), 6.99 (s, 1H), 6.92 (m, 1H), 6.90 (dd, J = 4.6, 1.3 Hz, 1H), 6.88 (s, 1H), 6.85 (t, J = 6.7 Hz, 1H), 6.78 (d, J = 6.0 Hz, 1H), 6.65 (d, J = 8.1 Hz, 1H), 6.60 (d, J = 8.1 Hz, 1H), 6.30 (dd, J = 11.4, 7.2 Hz, 3H), 2.94 (d, J = 6.1 Hz, 2H), 2.90 (d, J = 6.1 Hz, 2H).^{13}C NMR (101 MHz, $CDCl_3$) δ 172.38, 166.92, 166.61, 149.76, 148.57, 148.48, 148.41, 147.78, 147.38, 145.78, 144.10, 142.73, 142.68, 142.44, 142.30, 137.12, 137.05, 136.96, 134.05, 130.92, 130.82, 129.96, 129.74, 127.35, 125.39, 125.10, 123.86, 123.72, 122.48, 122.19, 121.91, 121.76, 120.71, 120.48, 118.71, 118.45, 114.66, 113.76, 37.13, 30.24. HRMS: Calcd. for $C_{40}H_{34}F_6IrN_4O_6P$ $[M–PF_6]^+$: 860.2209 Found: 860.2206.

Complex 12. (Yield: 85%) ^{1}H NMR (400 MHz, DMSO) δ 8.95 (s, 1H), 8.91 (d, J = 8.5 Hz, 1H), 8.79 (d, J = 8.5 Hz, 2H), 8.65 (s, 1H), 8.31 (s, 1H), 8.29 (s, 1H), 8.20 (dd, J = 5.1, 1.2 Hz, 1H), 8.04 (m, 2H), 7.99 (m, 2H), 7.54 (d, J = 5.9 Hz, 2H), 7.09 (m, 3H), 7.03 (d, J = 10.5 Hz, 2H), 6.69 (m, 2H), 6.57 (dd, J = 8.0, 2.0 Hz, 1H), 5.72 (m, 2H), 2.87 (s, 4H). ^{13}C NMR (101 MHz, DMSO) δ 172.20, 162.79, 162.71, 151.21, 150.02, 149.88, 149.82, 147.42, 146.17, 145.07, 143.46, 143.41, 143.21, 139.94, 139.09, 138.72, 134.19, 131.58, 127.82, 127.45, 127.13, 126.63, 124.50, 123.39, 123.23, 118.91, 117.10, 116.18, 115.93, 115.56, 113.89, 113.51, 113.50, 37.92, 30.43. HRMS: Calcd. for $C_{43}H_{29}F_{10}IrN_5O_3P$ $[M–PF_6]^+$: 932.1832 Found: 932.1813.

Complex 13. (Yield: 89%) ^{1}H NMR (400 MHz, $CDCl_3$) δ 8.46 (s, 1H), 8.36 (d, J = 4.3 Hz, 1H), 8.30 (m, 1H), 8.12 (m, 3H), 8.05 (d, J = 3.5 Hz, 2H), 7.98 (dd, J = 15.4,

7.6 Hz, 3H), 7.68 (dd, J = 8.4, 5.1 Hz, 1H), 7.55 (m, 2H), 7.47 (m, 1H), 7.15 (m, 5H), 7.02 (s, 1H), 6.88 (d, J = 8.8 Hz, 1H), 6.79 (t, J = 7.6 Hz, 3H), 6.68 (m, 1H), 6.57 (dd, J = 6.9, 3.9 Hz, 2H), 6.49 (d, J = 8.0 Hz, 1H), 6.43 (d, J = 7.9 Hz, 1H), 2.81 (s, 4H). ^{13}C NMR (101 MHz, $CDCl_3$) δ 172.10, 168.87, 168.58, 149.60, 149.56, 146.96, 146.76, 146.48, 145.73, 145.44, 144.71, 144.53, 143.72, 142.71, 142.19, 139.07, 138.66, 136.66, 134.02, 133.57, 132.51, 130.88, 130.58, 129.95, 129.77, 129.31, 128.19, 127.66, 126.49, 126.28, 126.10, 125.94, 125.93, 125.77, 125.73, 125.01, 124.42, 123.07, 122.79, 122.26, 122.02, 120.32, 118.56, 116.41, 116.00, 114.61, 113.61, 37.40, 30.28. HRMS: Calcd. for $C_{51}H_{37}F_6IrN_5O_3P$ $[M–PF_6]^+$: 960.2524 Found: 960.2505.

Complex 14. (Yield: 85%) ^{1}H NMR (400 MHz, $CDCl_3$) δ 8.59 (d, J = 8.2 Hz, 1H), 8.51 (d, J = 8.6 Hz, 1H), 8.15 (t, J = 5.3 Hz, 2H), 7.82 (d, J = 8.3 Hz, 2H), 7.64 (m, 2H), 7.63 (m, 2H), 7.31 (d, J = 5.2 Hz, 2H), 7.17 (d, J = 3.1 Hz, 2H), 7.05 (d, J = 8.2 Hz, 1H), 7.01 (t, J = 7.3 Hz, 3H), 6.90 (d, J = 7.5 Hz, 2H), 6.87 (d, J = 3.7 Hz, 2H), 6.83 (m, 2H), 6.80 (d, J = 7.2 Hz, 2H), 6.32 (d, J = 7.5 Hz, 2H), 3.81 (m, 2H), 2.98 (t, J = 6.8 Hz, 2H), 2.20 (s, 3H), 2.19 (s, 3H). ^{13}C NMR (101 MHz, $CDCl_3$) δ 167.47, 167.46, 165.71, 149.60, 148.74, 148.50, 148.10, 147.68, 145.85, 142.74, 142.66, 140.87, 139.60, 138.56, 137.02, 136.86, 136.52, 134.51, 130.90, 129.75, 129.70, 128.42, 126.68, 126.57, 125.59, 125.37, 123.69, 123.65, 123.02, 122.62, 122.43, 122.27, 121.71, 118.44, 39.49, 33.44, 19.70, 19.63. HRMS: Calcd. for $C_{47}H_{37}F_6IrN_5O_5P$ $[M–PF_6]^+$: 944.2421 Found: 944.2453.

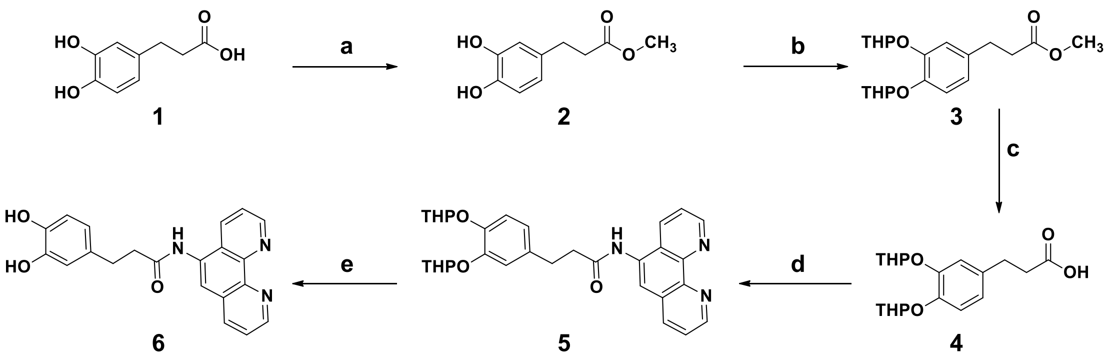

Scheme S1. Synthesis of ligand **6** reagents and conditions: a) $MeOH/H_2SO_4$, reflux, overnight, Yield = 97%; b) THP, PPTS/DCM, Yield = 95%; c) LiOH, THF/H_2O (1:1), 2 h, 0 ℃ to RT, Yield = 90%; d) 1,10-phenanthrolin-5-amine, DMAP, EDCI, DCM, Yield = 75%; e) PPTS/EtOH, 50 ℃, Yield = 95%.

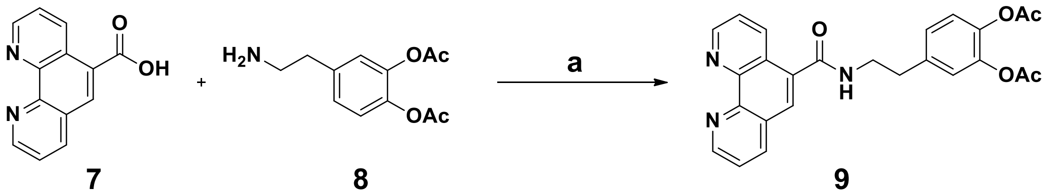

Scheme S2. Synthesis of ligand **9** reagents and conditions: a) Et_3N, EDCI, HOBt, DCM, Yield = 62%.

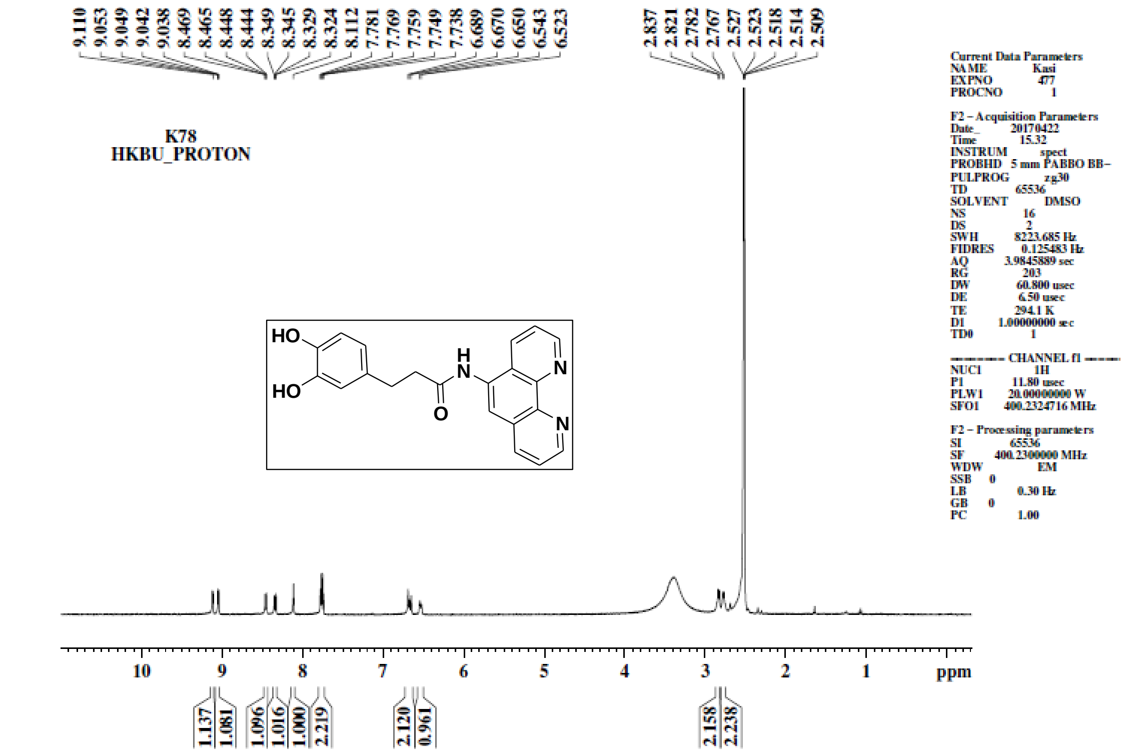

Fig. S1a ^{1}H NMR spectrum of compound **6**.

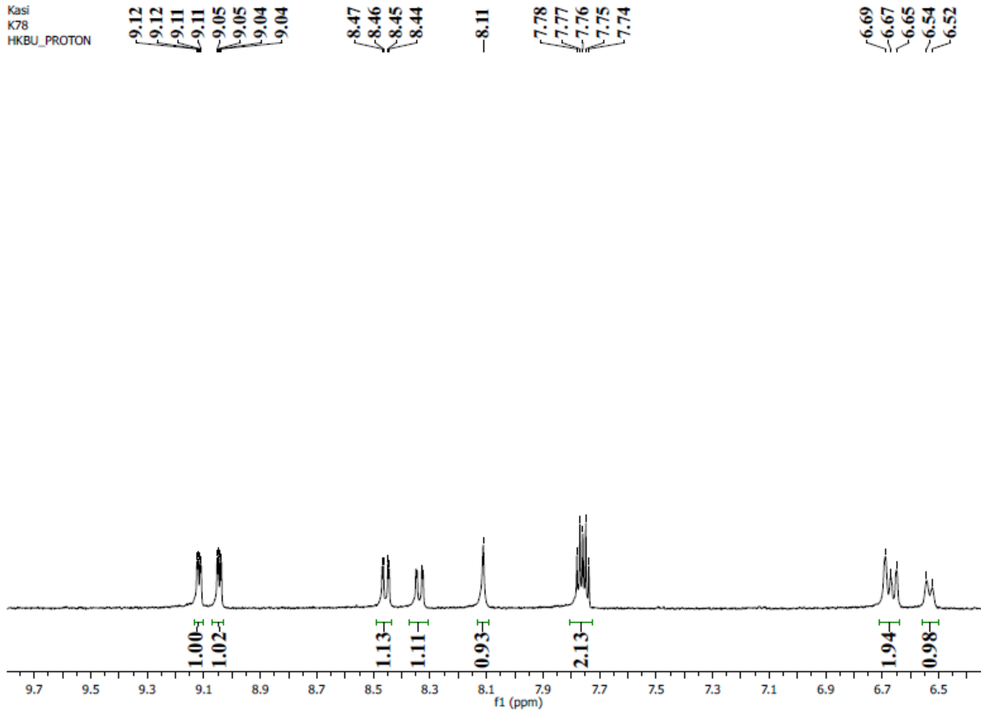

Figure S1b. Expanded ^{1}H NMR spectrum of ligand **6**.

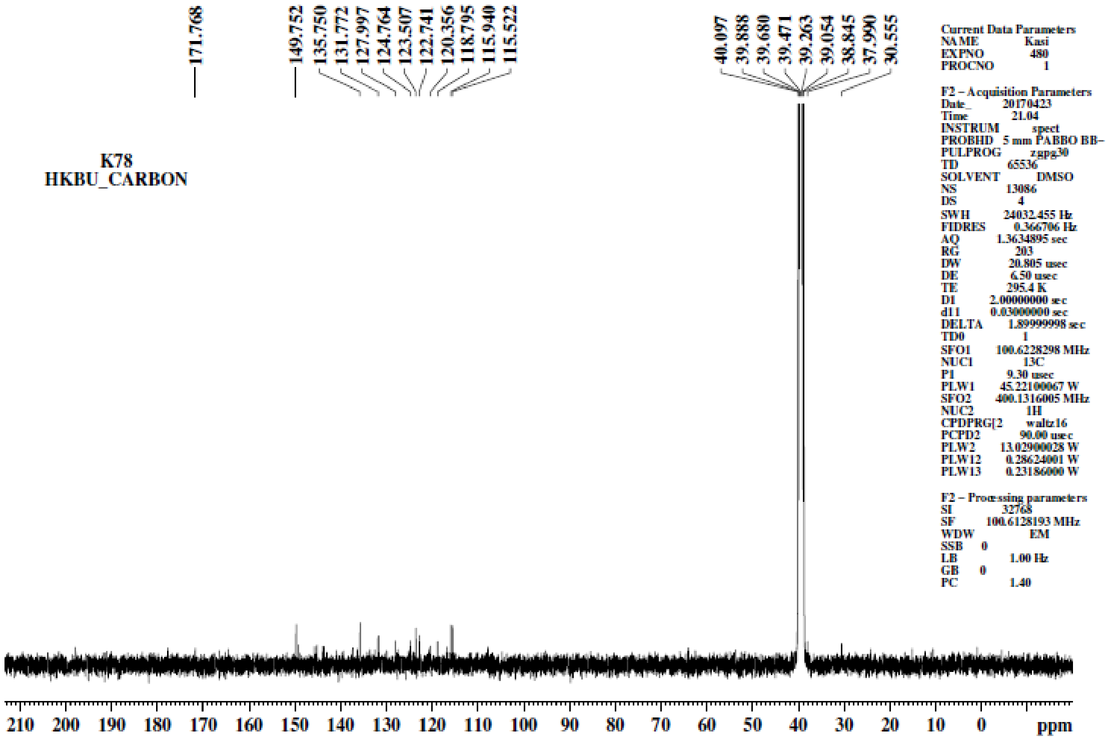

Fig. S2 ^{13}C NMR spectrum of compound **6**.

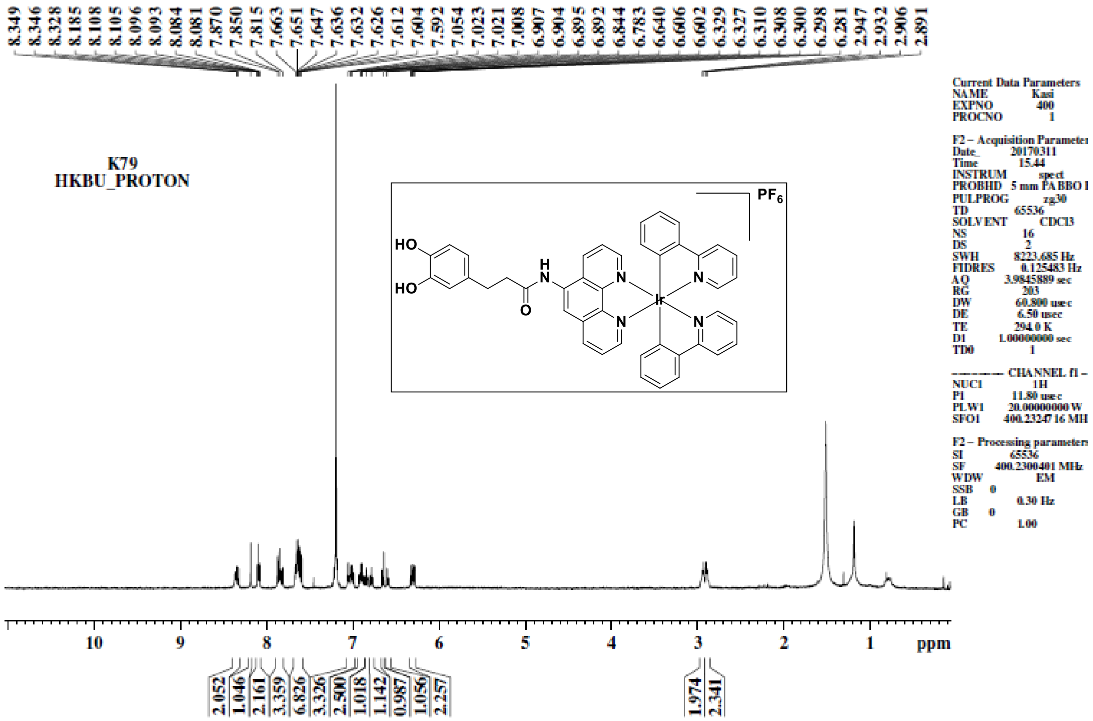

Fig. S3a ^{1}H NMR spectrum of complex **11**.

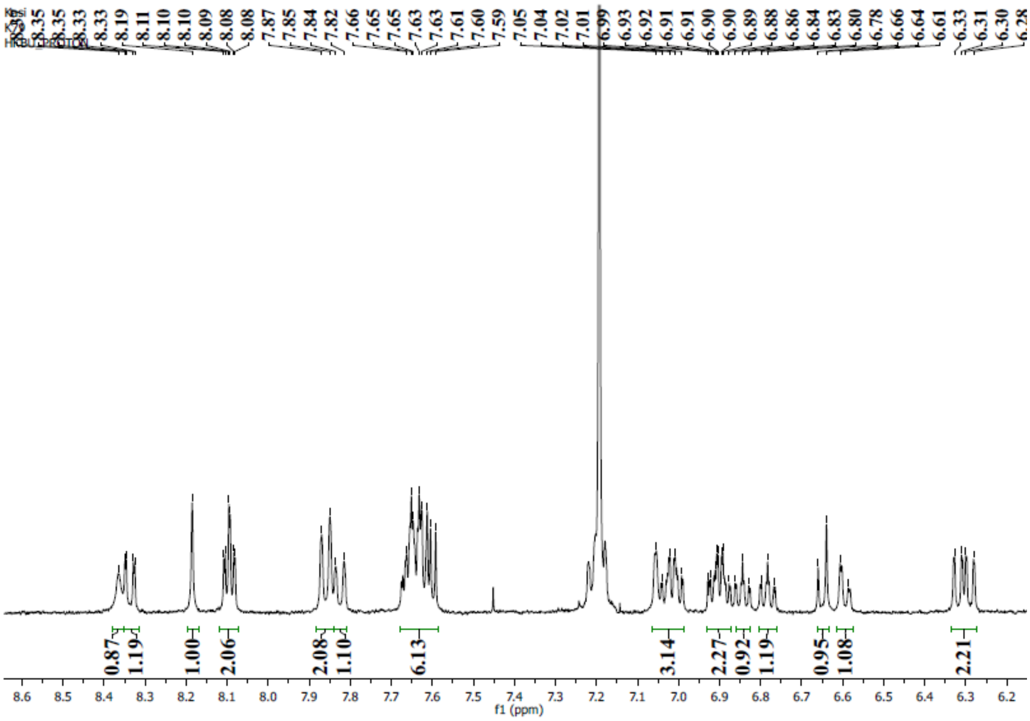

Figure S3b. Expanded ^{1}H NMR spectrum of complex **11**.

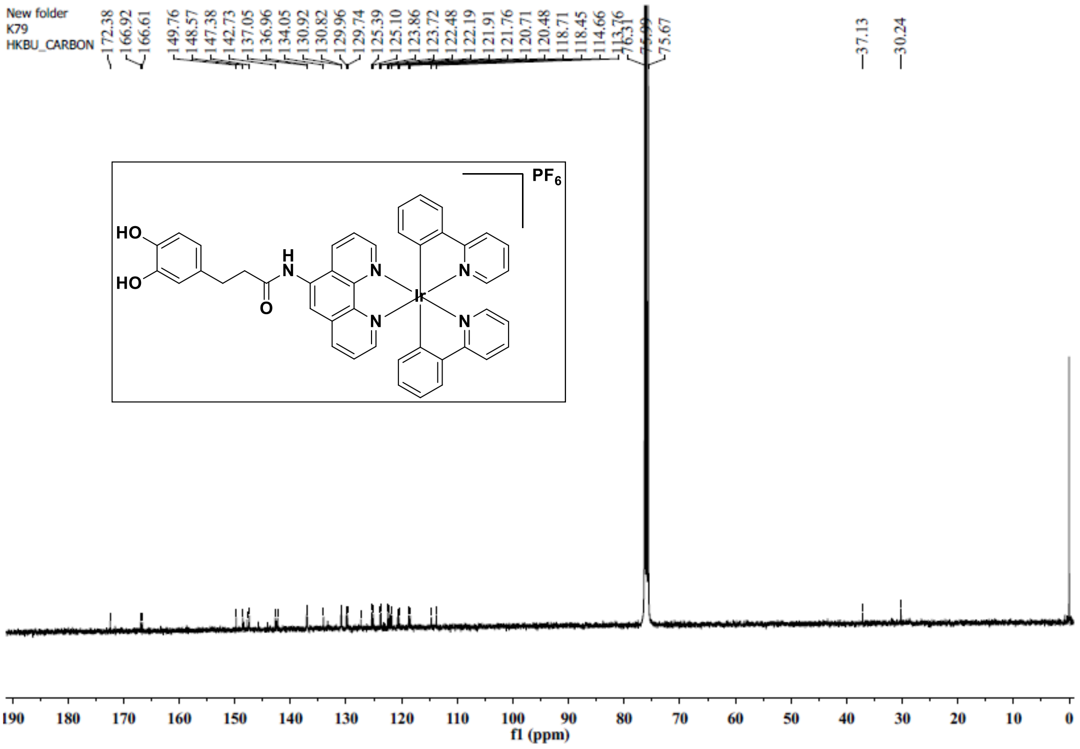

Fig. S4 ^{13}C NMR spectrum of complex **11**.

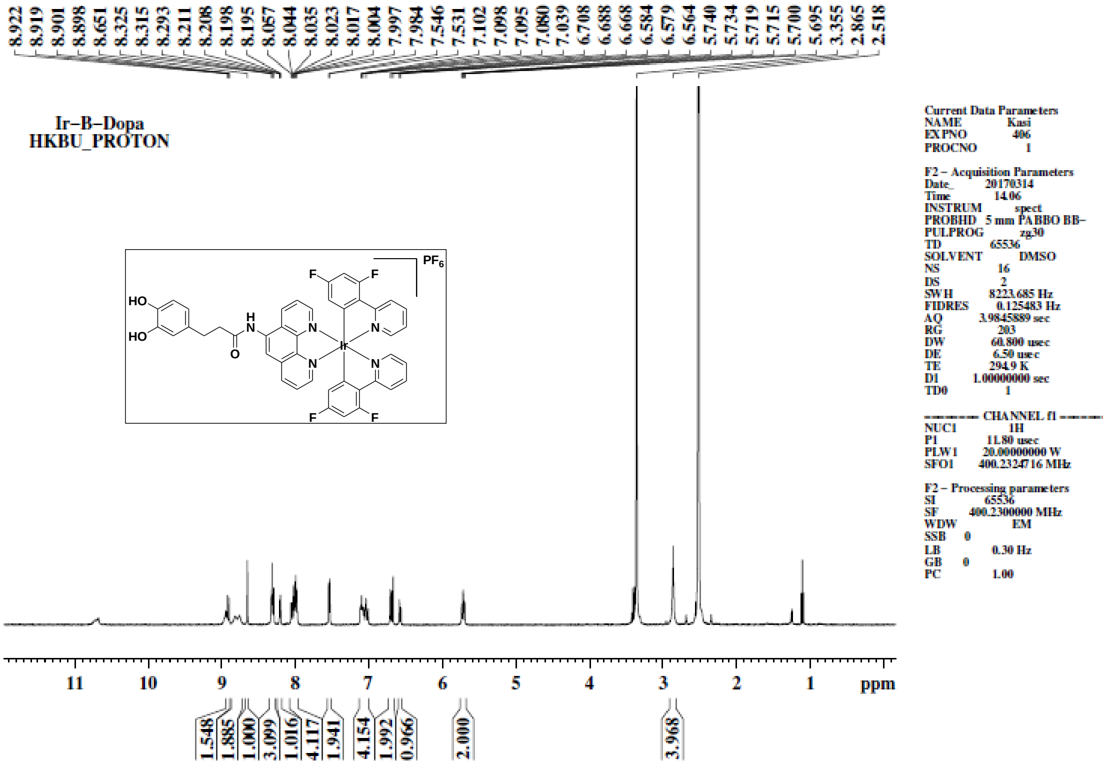

Fig. S5a ^{1}H NMR spectrum of complex **12**.

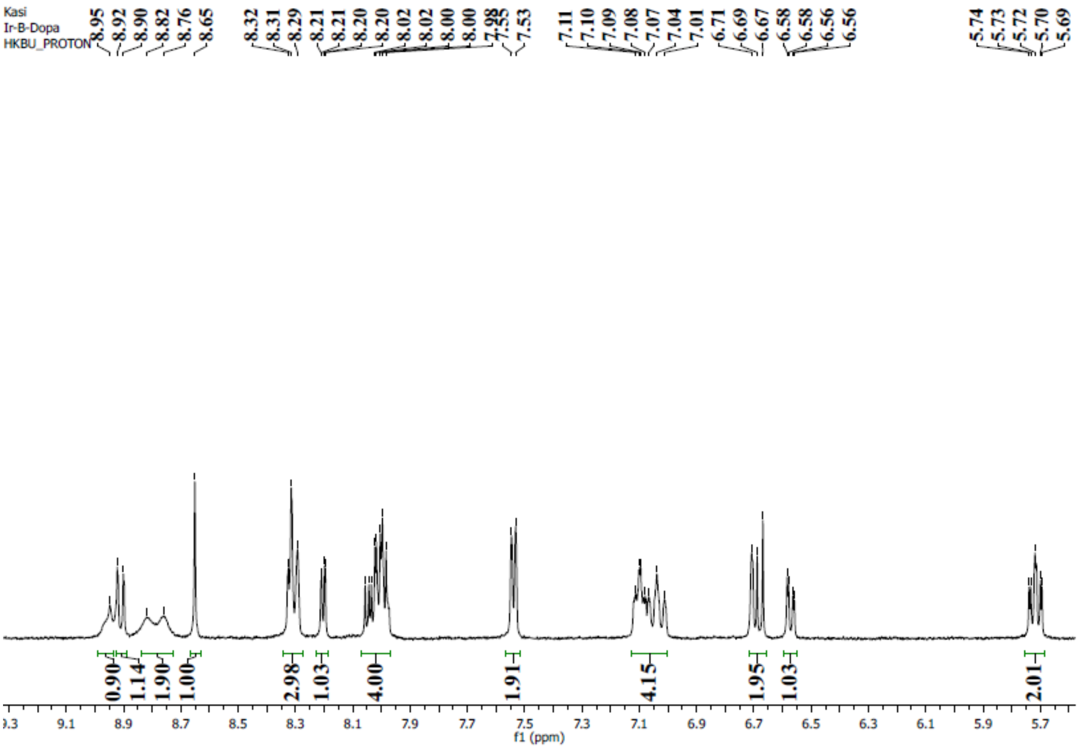

Figure S5b. Expanded ^{1}H NMR spectrum of complex **12**.

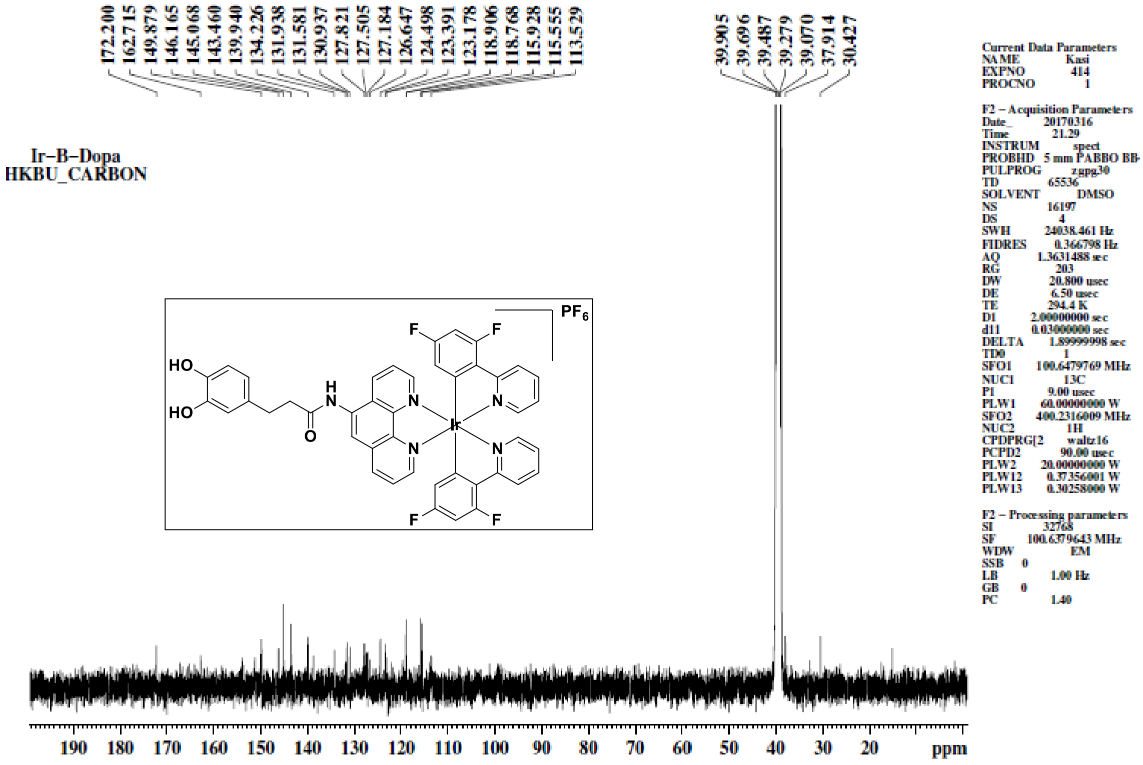

Fig. S6 ^{13}C NMR spectrum of complex **12**.

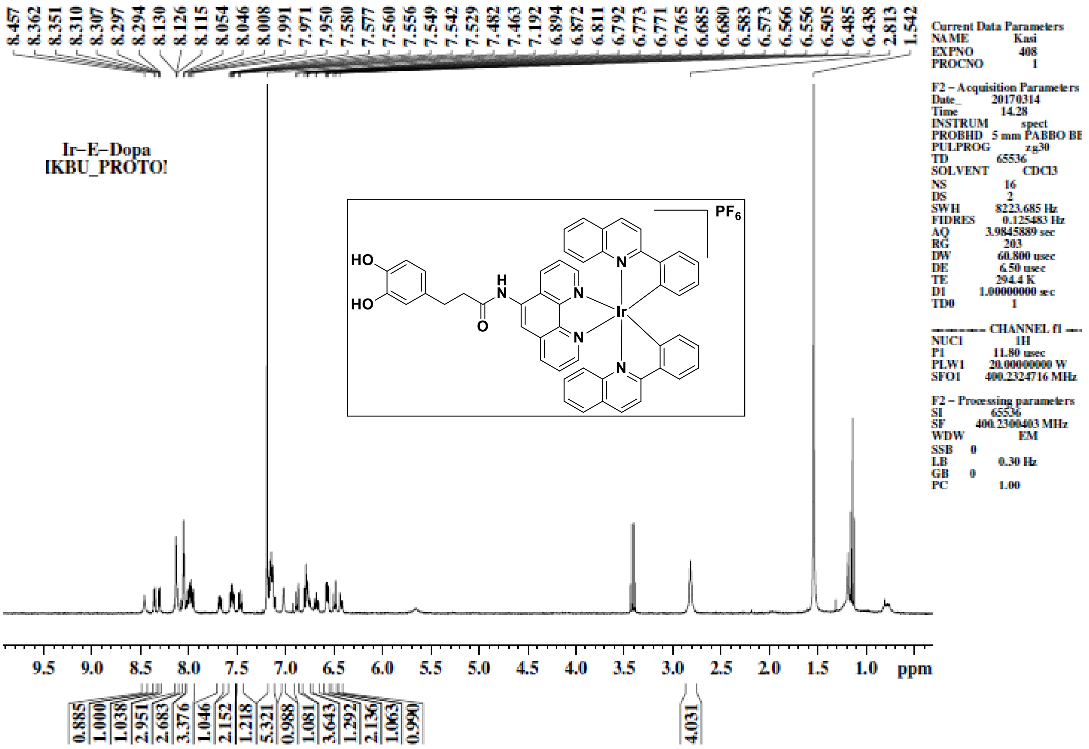

Fig. S7a ^{1}H NMR spectrum of complex **13**.

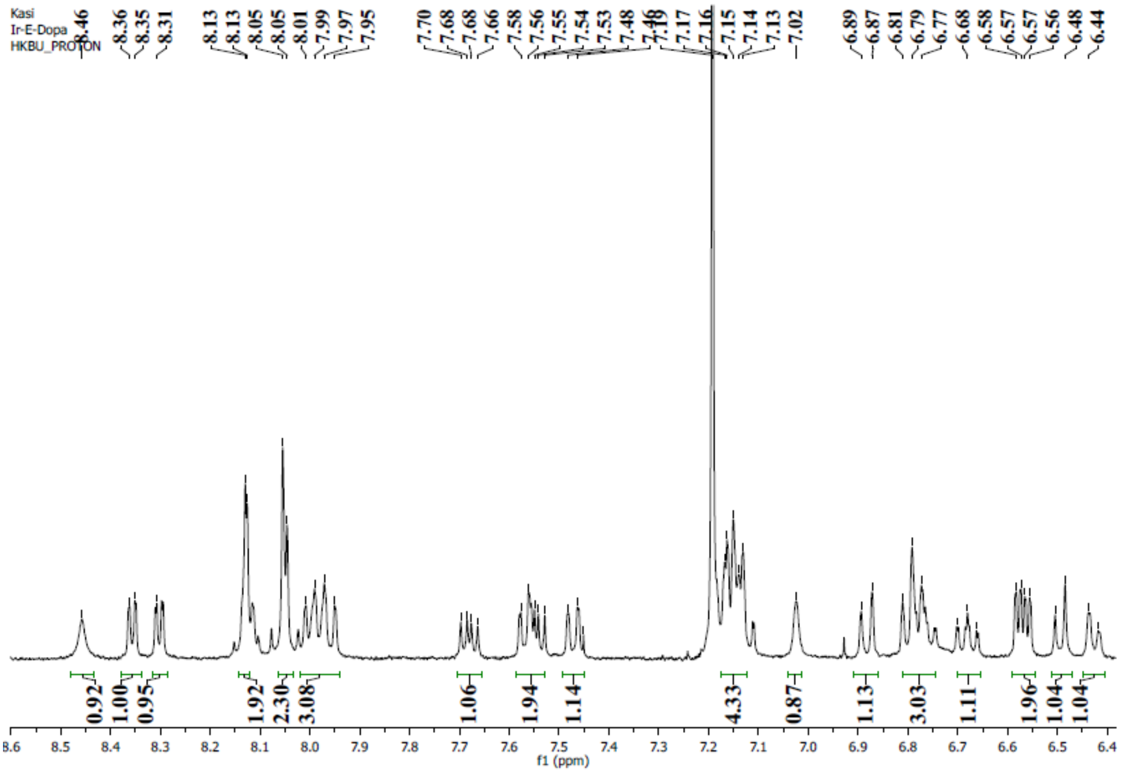

Figure S7b. Expanded ^{1}H NMR spectrum of complex **13**.

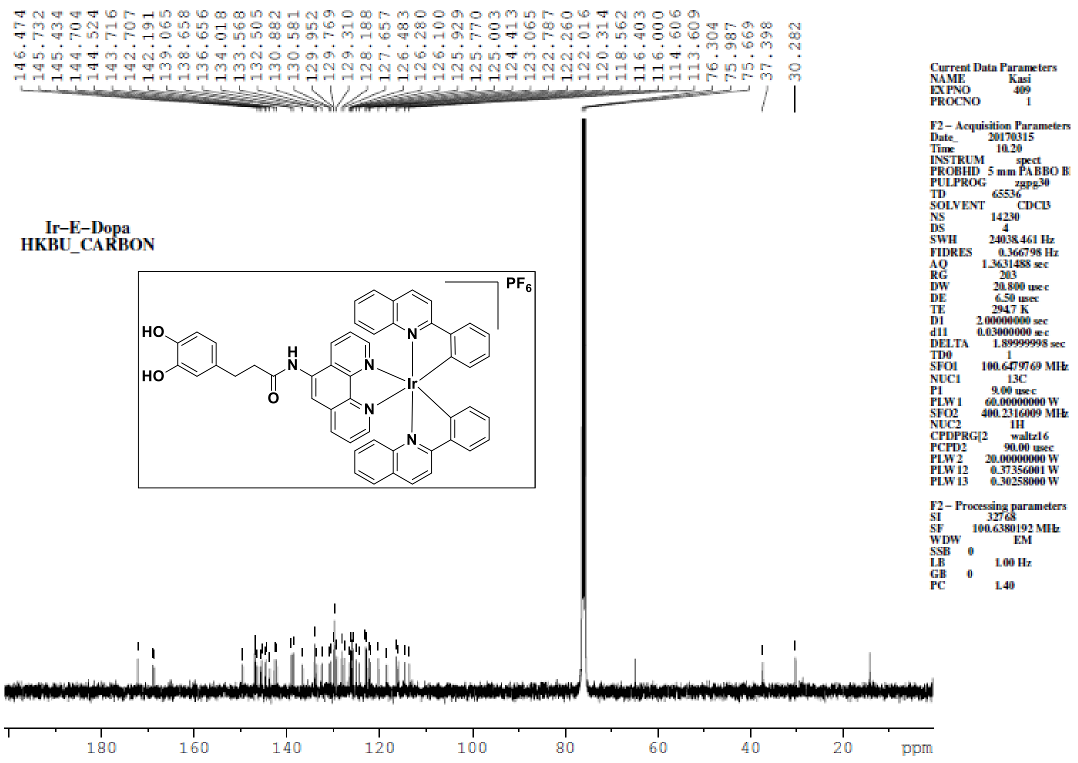

Fig. S8 ^{13}C NMR spectrum of complex **13**.

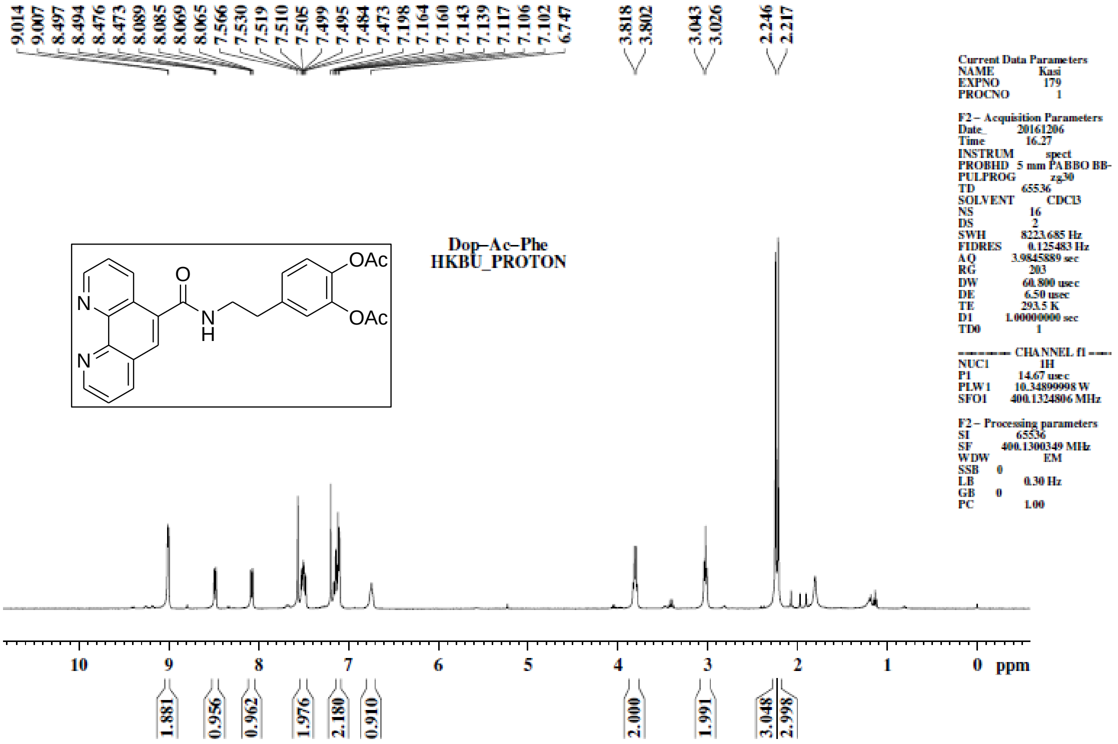

Fig. S9a ^{1}H NMR spectrum of Ligand **9**.

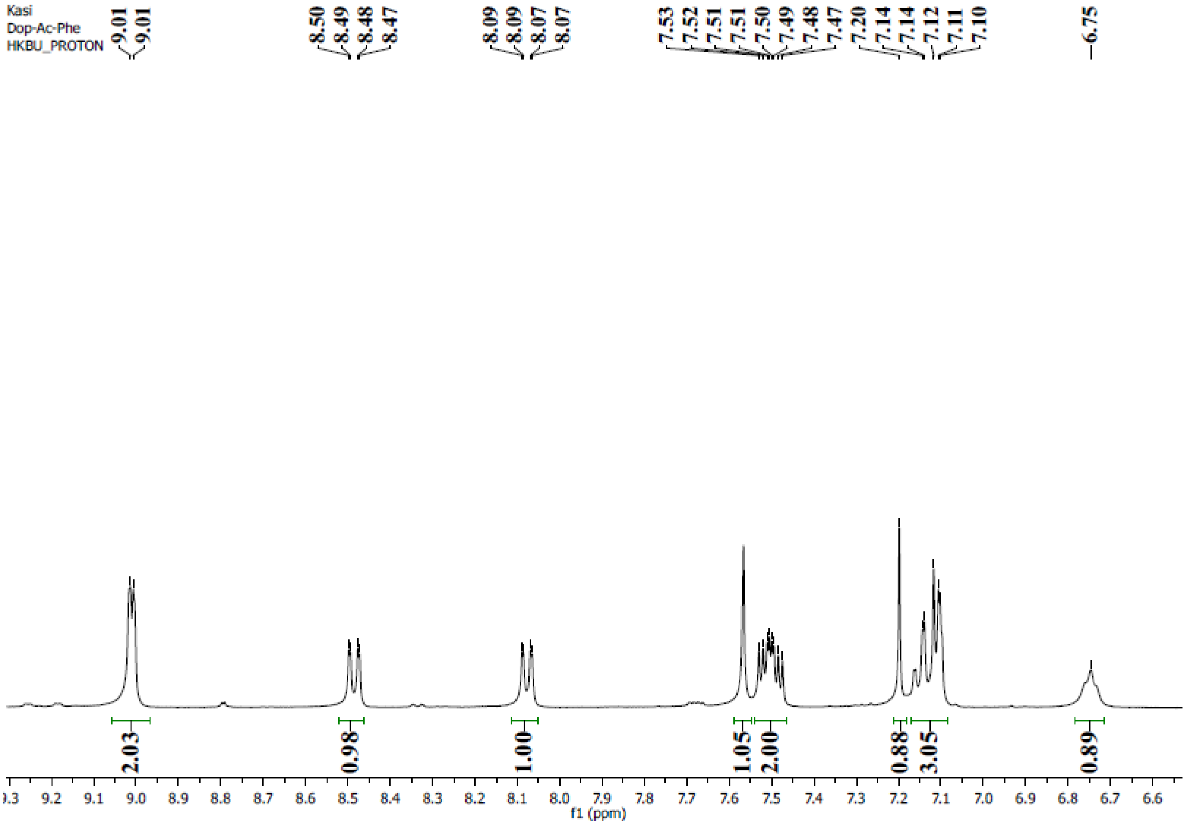

Figure S9b. Expanded ^{1}H NMR spectrum of Ligand **9**.

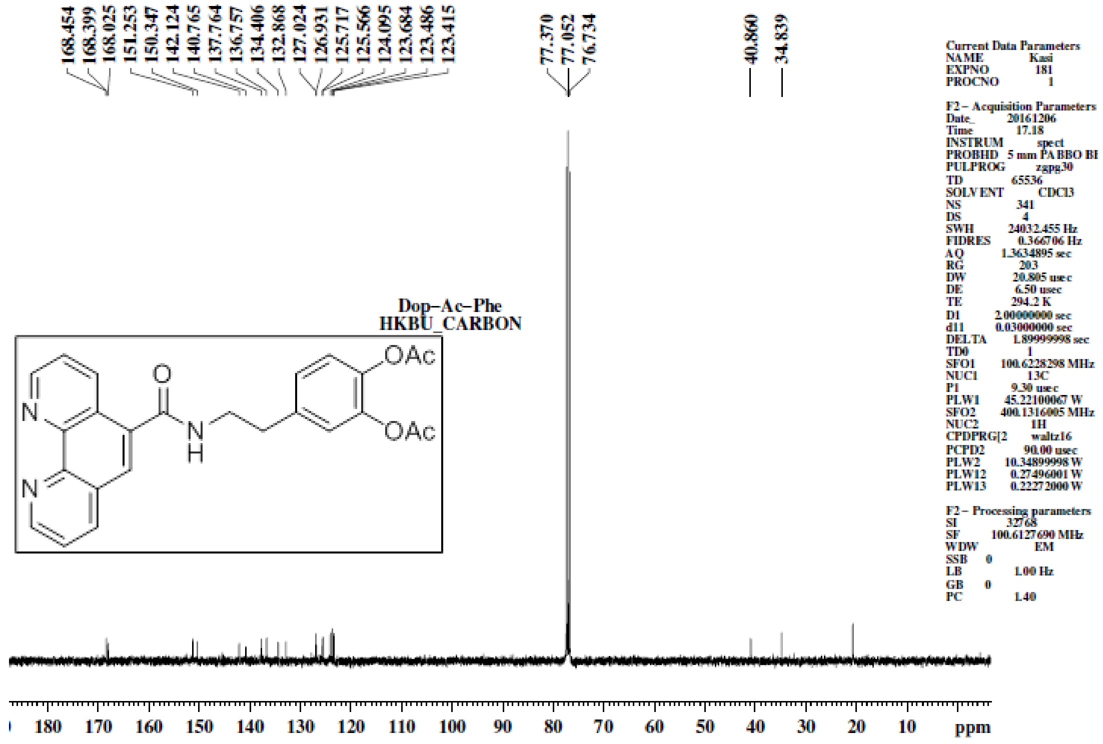

Fig. S10 ^{13}C NMR spectrum of Ligand **9**.

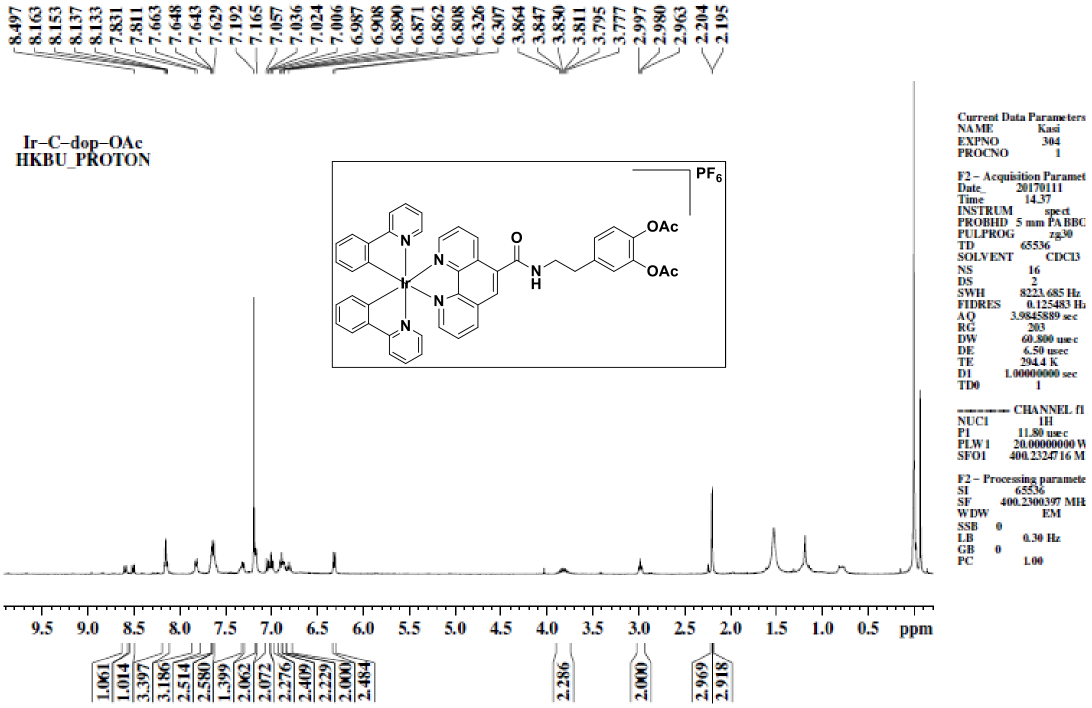

Fig. S11a^{1}H NMR spectrum of complex **14**.

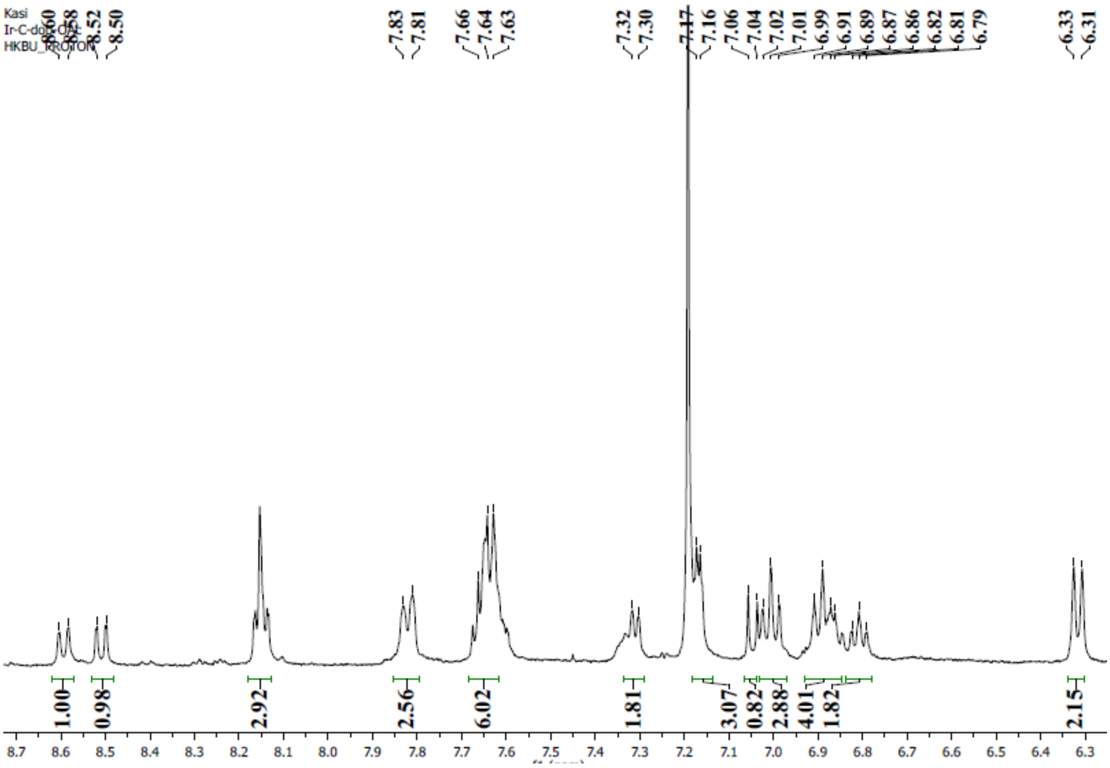

Figure S11b. Expanded ^{1}H NMR spectrum of complex **14**.

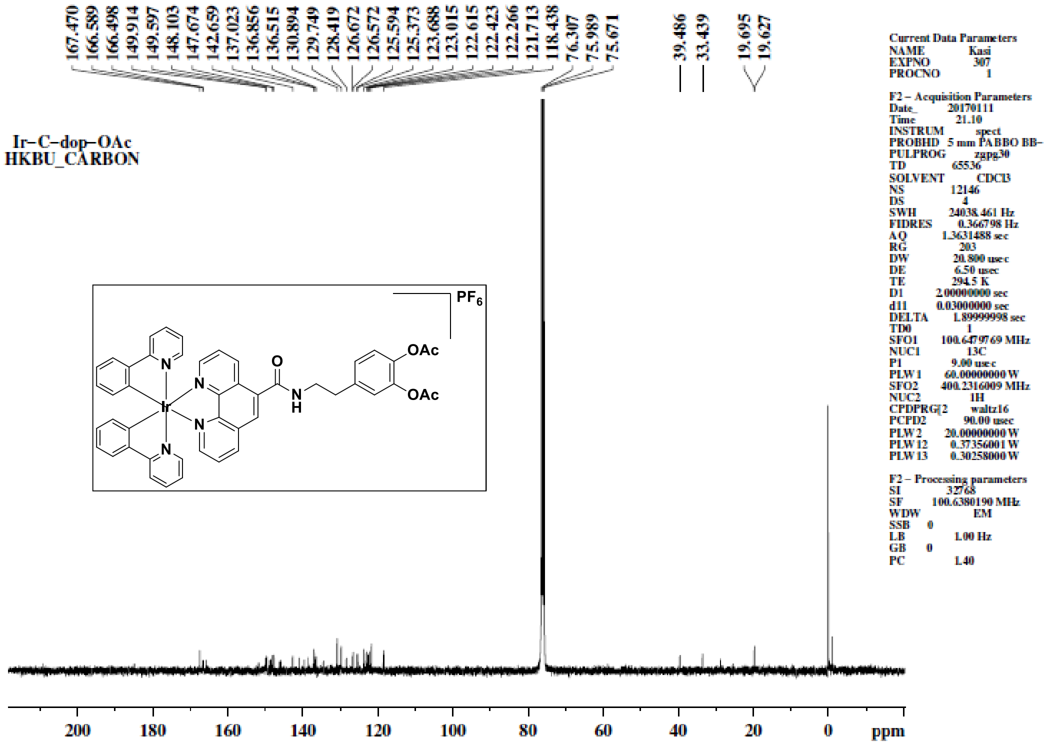

Fig. S12 ^{13}C NMR spectrum of complex **14**.

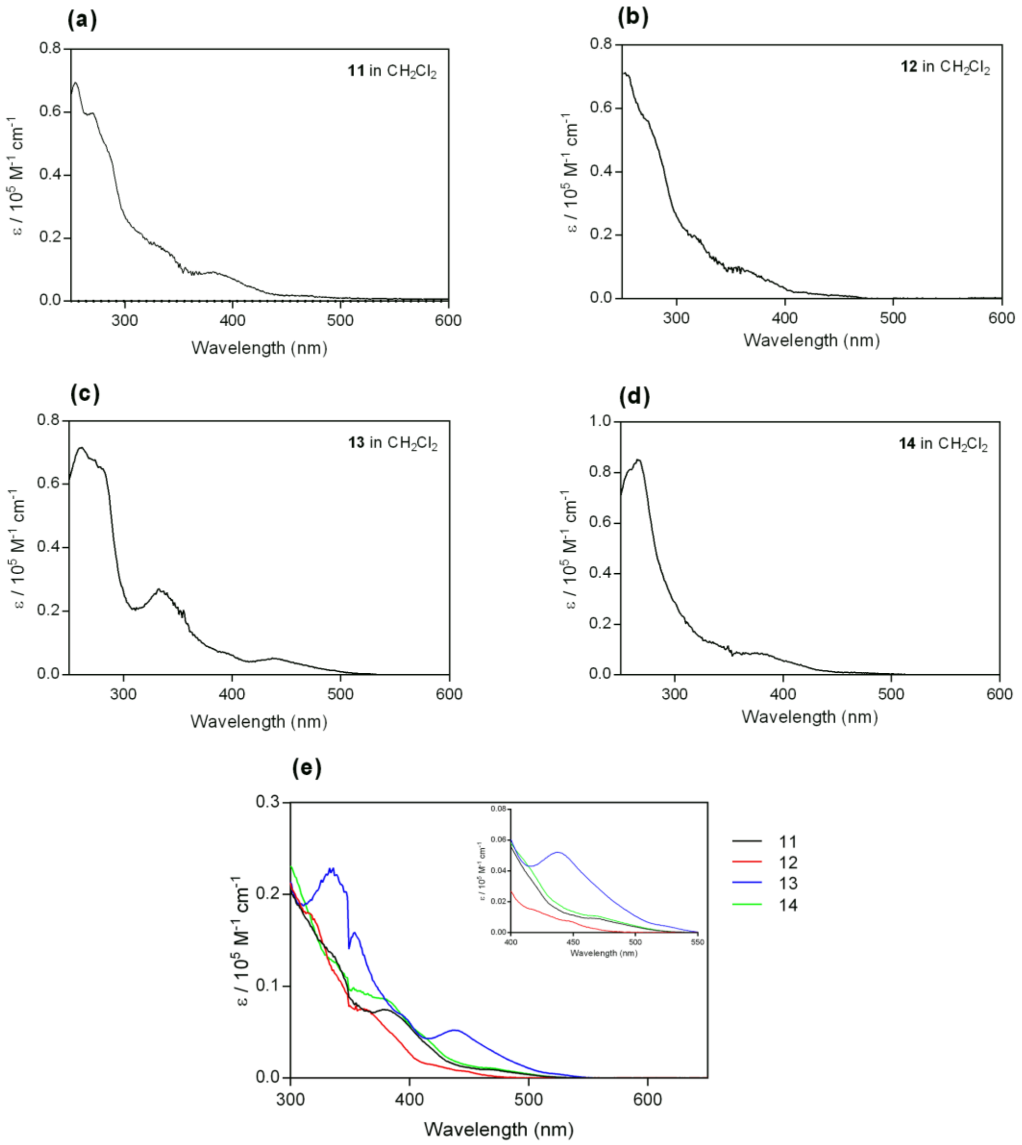

Fig. S13 Absorption spectra of 10 μM of complexes (a) **11**, (b) **12**, (c) **13** and (d) **14** in degassed CH_2Cl_2 at 298 K, (e) UV-Vis spectrum of complexes **11**−**14** (10 μM) in degassed CH_2Cl_2 at 298 K.

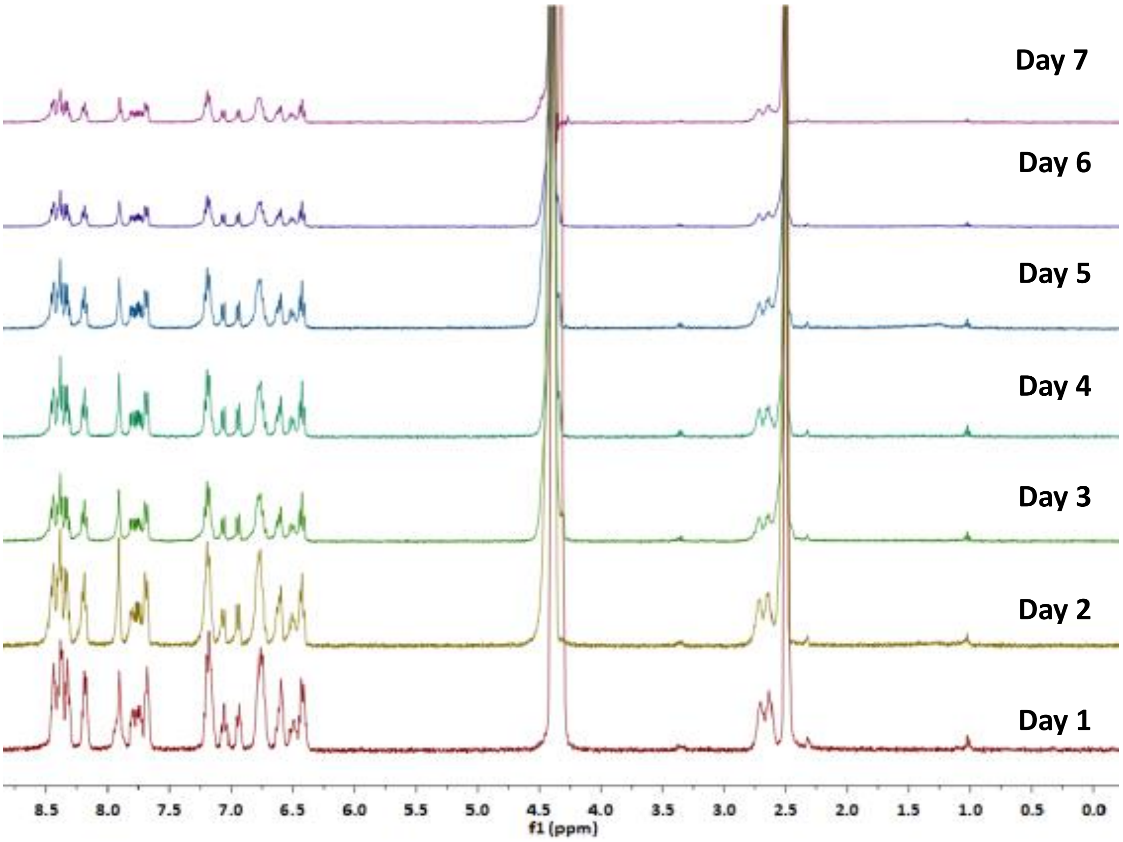

Fig. S14 ^{1}H NMR spectra of complex **13** in DMSO-d_6/ D_2O (9:1) at 298 K over 7 days.

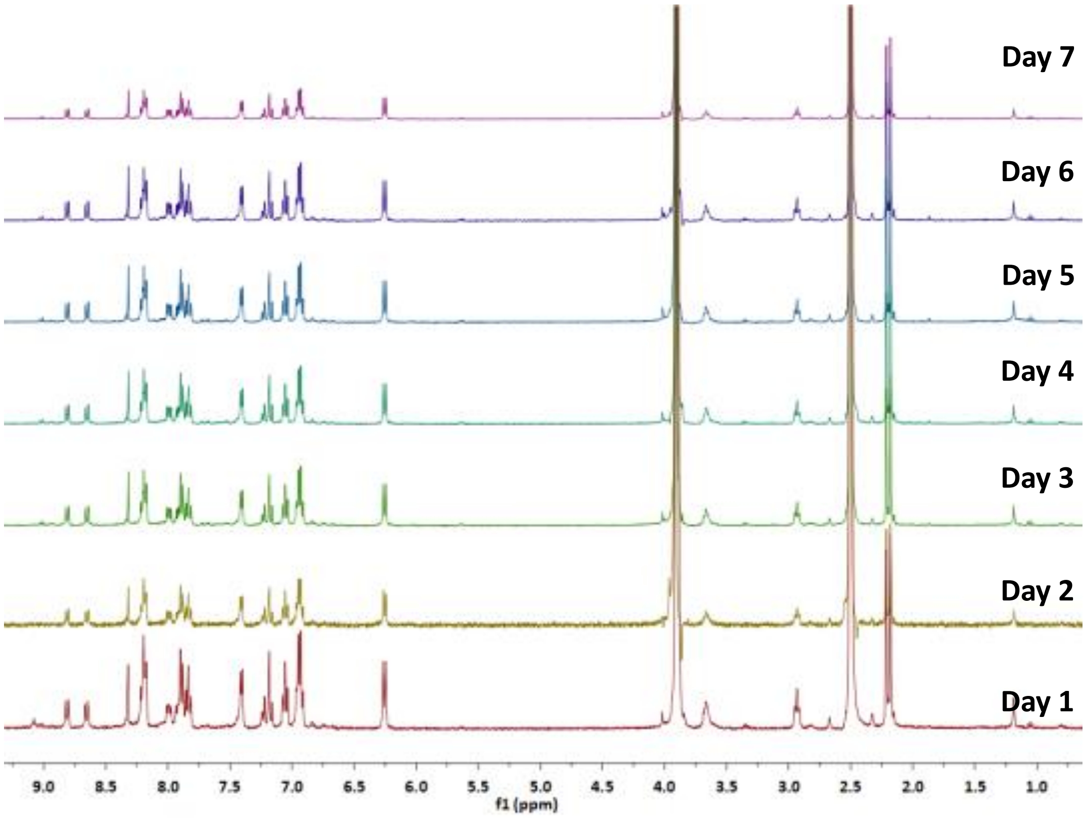

Fig. S15 ^{1}H NMR spectra of complex **14** in DMSO-d_6/ D_2O (9:1) at 298 K over 7 days.

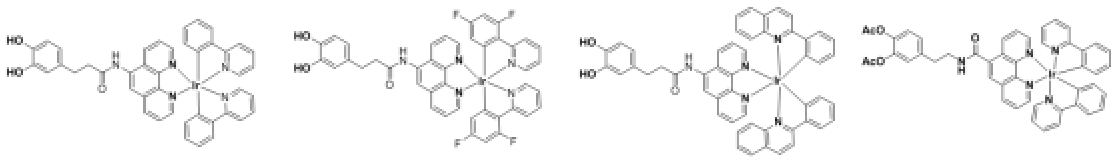

11 **12** **13** **14**

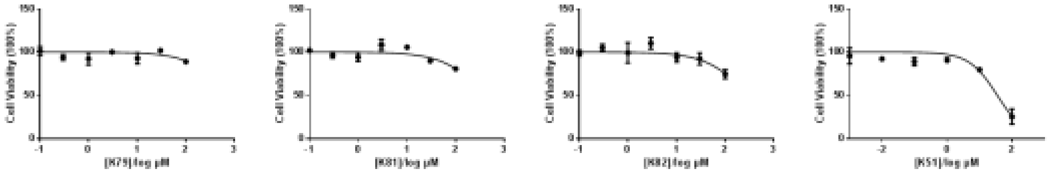

Fig. S16 A549 cells were treated with different concentration of complexes for 48 h. Complexes **11**, **12** and **13** inhibited cell viability of A549 cells with IC_{50} value > 100 μM. Complex **14** exhibited an IC_{50} value of 70.79 μM.

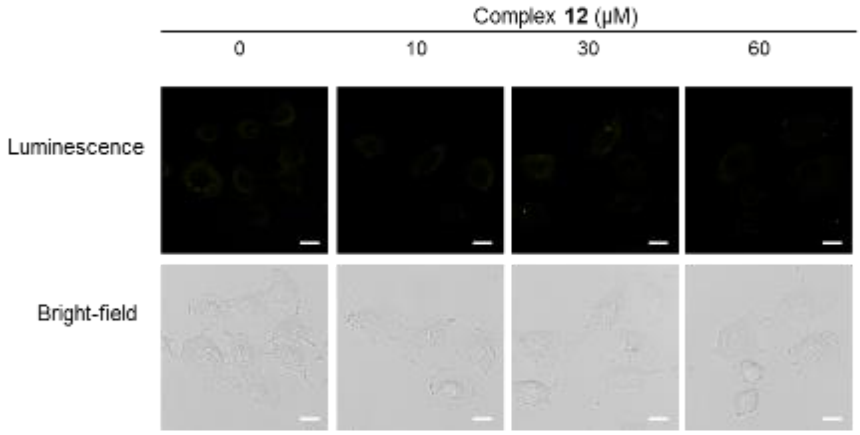

Fig. S17 A549 cells were stained by different concentrations of complex **12** (0, 10, 30 and 60 μM) for 1 h. Scale bar = 15 μm.

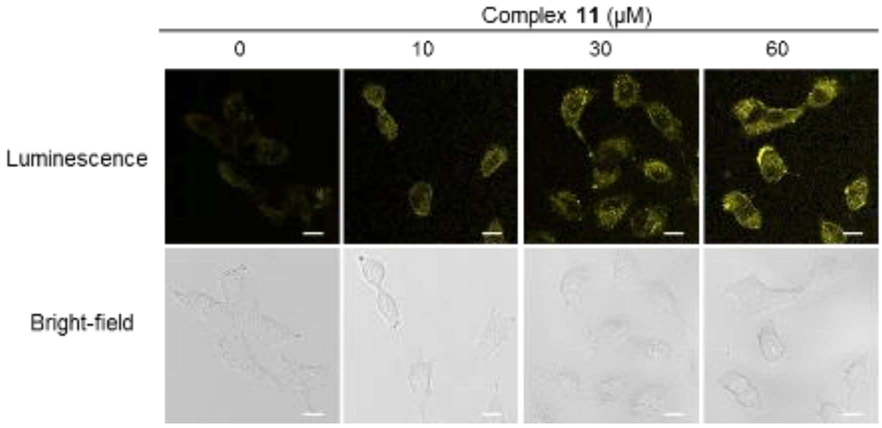

Fig. S18 A549 cells were stained by different concentrations of complex **11** (0, 10, 30 and 60 μM) for 1 h. Scale bar = 15 μm.

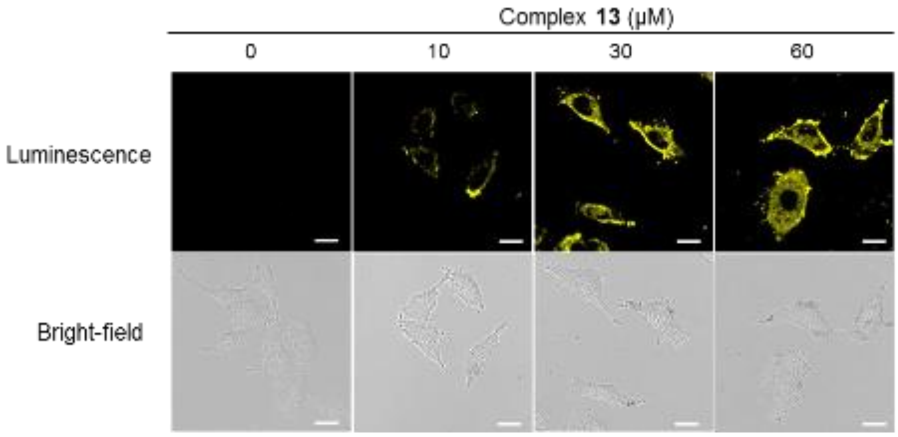

Fig. S19 A549 cells were stained by different concentrations of complex **13** (0, 10, 30 and 60 μM) for 1 h. Scale bar = 15 μm.

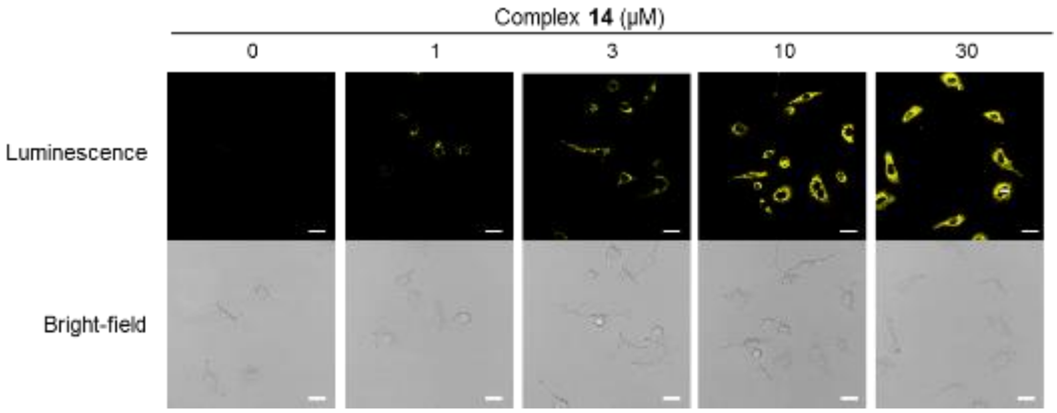

Fig. S20 A549 cells were stained by different concentrations of complex **14** (0, 1, 3, 10 and 30 μM) for 1 h. Scale bar = 30 μm.

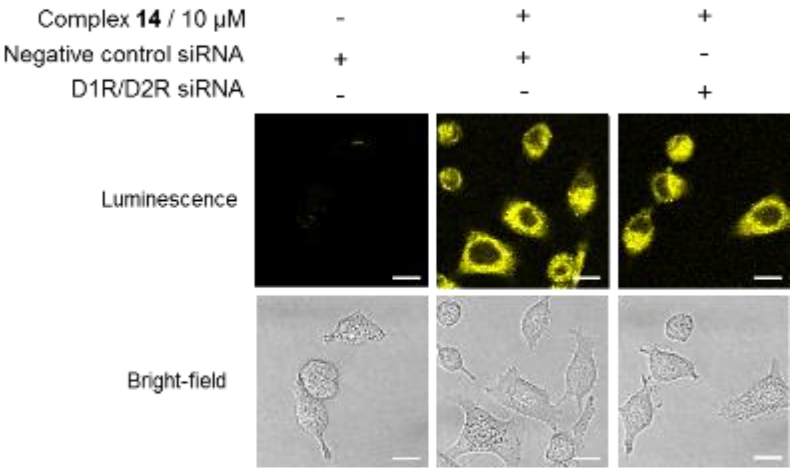

Fig. S21 Luminescence and bright-field images of complex **14**-stained A549 cells with or without D1R/D2R knockdown using siRNA. Scale bar = 15 μm.

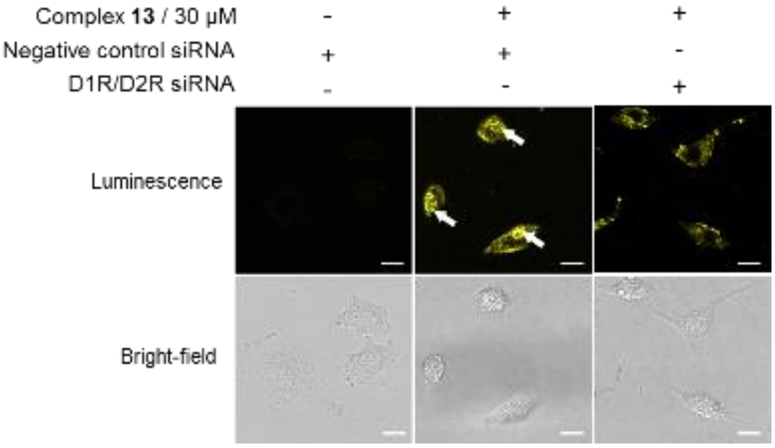

Fig. S22 Luminescence and bright-field images of complex **13**-stained A549 cells with or without D1R/D2R knockdown using siRNA. A549 cells were stained with complex **13** (30 μM) or DMSO for 180 min. Scale bar = 15 μm.

Table S1. Photophysical properties of iridium(III) complexes **11**–**14**.

Complex	Quantum yield	λ_{em} / nm	Lifetime / μs	UV/Vis absorption λ_{abs} / nm (ε / M^{-1} cm^{-1})
**11**	0.196	582	4.36	251 (0.27×10^5), 377 (0.074×10^5)
**12**	-	-	-	246 (0.28×10^5), 279 (0.26×10^5), 357 (0.074×10^5)
**13**	0.245	558	4.61	255 (0.27×10^5), 280 (0.26×10^5), 331 (0.23×10^5), 446 (0.048×10^5)
**14**	0.135	586	4.65	265 (0.27×10^5), 374 (0.087×10^5)

References

1. Narayanan J, Hayakawa Y, Fan J, Kirk KL. Bioorganic Chemistry 31, 191-197 (2003).
2. Hou B-Y, Zheng Q-Y, Wang D-X, Wang M-X. Tetrahedron 63, 10801-10808 (2007).
3. Elmes RBP, Orange KN, Cloonan SM, Williams DC, Journal of the American Chemical Society 133, 15862-15865 (2011).
